# *Lactobacillus* shapes LPS-reservoir modules within the gut microbiota to mitigate atrial fibrillation

**DOI:** 10.1128/mbio.00741-26

**Published:** 2026-05-29

**Authors:** Xinyuan Wang, Xiwen Wang, Song Xu, Jia Zheng, Yan Pang, Tingting Cheng, Ruiqin Han, Wei Zhao, Zhiyong Huang

**Affiliations:** 1 Tianjin Key Laboratory for Industrial Biological Systems and Bioprocessing Engineering, Tianjin Institute of Industrial Biotechnology, Chinese Academy of Sciences, Tianjin, China; 2 National Technology Innovation Center of Synthetic Biology 658577 , Tianjin, China; 3 University of Chinese Academy of Sciences 74519, Beijing, China; 4 State Key Laboratory of Biocatalysis and Enzyme Engineering, School of Life Sciences, Hubei University, Wuhan, Hubei, China; 5 The First Central Clinical School, Tianjin Medical University, Tianjin, China; 6 Department of Cardiology, Tianjin First Center Hospital, Tianjin, China; 7 College of Bioengineering,, Tianjin University of Science and Technology, Tianjin, China; The University of Tennessee Knoxville, Knoxville, Tennessee, USA

**Keywords:** gut microbiota, cardiovascular, atrial fibrillation, *Lactobacillus*, lipopolysaccharides

## Abstract

Atrial fibrillation (AF) is the most prevalent clinical arrhythmia, associated with numerous complications, elevated morbidity and mortality rates, and limited therapeutic interventions, which have been confirmed to be related to the intestinal microbiota. However, there is currently limited research on the interaction between gut microbiota and AF. Herein, we integrated the largest Chinese gut microbiota cohort (*n* = 355) related to AF to date and identified a marked microbial imbalance in AF patients, characterized primarily by a significant reduction in *Lactobacillus* abundance. Using a rat model, we validated the role of *Lactobacillus* in lowering intestinal lipopolysaccharide (LPS) levels and alleviating host inflammation and AF-related symptoms. Mechanistically, microbial network analysis revealed that *Lactobacillus* promotes the formation of “LPS-reservoir modules,” enhancing microbial community stability and reducing free LPS levels, thereby mitigating AF pathogenesis. These findings offer novel insights into the complex interplay among gut microbiota, LPS, and cardiovascular diseases.

**IMPORTANCE:**

AF is a common heart rhythm disorder with limited treatment options. While emerging evidence links AF to the gut microbiome, the exact mechanisms remain poorly understood. Here, we analyzed a large cohort of AF patients and discovered a significant depletion of *Lactobacillus* in their gut microbiota. Using an animal model, we demonstrated that replenishing *Lactobacillus* reduces systemic inflammation and alleviates AF symptoms. Mechanistically, *Lactobacillus* promotes the formation of stable microbial networks. This enhanced stability prevents massive bacterial death and turnover, thereby reducing the release of free LPS into the environment. This work is highly significant as it decodes a vital link between gut stability and cardiovascular health, suggesting that *Lactobacillus*-based probiotics could serve as an innovative strategy for managing AF.

## INTRODUCTION

Atrial fibrillation (AF) is the most prevalent and sustained cardiac arrhythmia, resulting from hemodynamic disturbances, atrioventricular dyssynchrony, and atrial structural remodeling (1). In recent decades, the incidence of AF has surged, with global prevalence rising from 28.3 million in 1990 to 45.6 million in 2010, and further to a staggering 59.7 million cases (2). Consequently, there is an urgent need to develop more effective treatment strategies for AF. Currently, the treatments for AF include surgical interventions such as catheter ablation and external pacing (3). However, due to its complex pathophysiology and the diverse range of associated complications, AF patients continue to experience high rates of rehospitalization (4). Therefore, identifying novel and effective interventions is imperative.

Recent studies have increasingly implicated the gut microbiota in cardiovascular health (5). For example, Chen et al. discovered that intestinal butyrate producers can confer cardioprotection (6). Matthew et al. found that *Lactobacillus reuteri* can alleviate myocardial infarction (7). In the context of AF, the role of the gut microbiota has also been discovered. Fang et al. performed fecal microbiota transplantation (FMT) from AF-model mice into microbiota-depleted recipients, thereby substantiating a causal role for the microbiome in AF pathogenesis (8). Zhang et al. reported that dysbiosis promotes age-related AF by elevating circulating LPS levels (4). Conversely, several commensals exert protective effects on AF. Luo et al. found that *Akkermansia muciniphila* attenuates cold-induced AF via modulation of TMAO-triggered cardiac autophagy (9). Zhang et al. revealed that *Bacteroides fragilis* prevents age-related AF in rats by orchestrating regulatory T-cell-mediated anti-inflammatory responses (10). These findings highlight the therapeutic potential of microbiota-targeted interventions for AF.

Although some progress has been made in the above-mentioned studies, research on the relationship between the gut microbiota and AF still faces significant challenges. On one hand, studies examining this association are scarce (11), and those that do exist are hampered by seriously limited cohort sizes (12–14). As a result, the link between gut microbial communities and AF remains incompletely defined, and our understanding of the underlying mechanisms is still superficial. On the other hand, most current investigations have focused on individual molecular factors, lacking a system-level perspective of the intestinal ecosystem. For instance, while the pathogenic role of LPS is well-characterized, the ecological drivers governing its pathological accumulation remain elusive. Unlike secreted metabolites such as short-chain fatty acids (15), LPS is an integral component of the bacterial outer membrane, typically released upon cell lysis or turnover (16). Consequently, fluctuations in bacterial abundance alone cannot fully account for the elevation of free LPS levels; rather, ecosystem instability likely plays a pivotal role in this process. Therefore, a more comprehensive, ecosystem-based study of the interactions between the gut microbiota and AF is urgently needed.

To overcome these limitations and account for dietary differences among populations in different countries, we integrated multiple Chinese AF-related gut microbiota cohorts and established the largest Chinese cohort of AF to date. We characterized the compositional and functional alterations in the fecal microbiota of AF patients and, using machine-learning algorithms, identified *Lactobacillus* as a key genus with potential to ameliorate AF. Adopting a micro-ecosystem perspective, we employed molecular ecological network analysis to evaluate ecological stability and, in conjunction with modularity analysis, investigated the regulatory mechanisms driving LPS accumulation. This comprehensive, system-level approach enables us to elucidate the complex microbial interactions contributing to AF pathogenesis.

## MATERIALS AND METHODS

### Study design, subject recruitment, and sample collection

This study integrated a total of four distinct Chinese cohorts. Specifically, the Tianjin cohort was recruited and established in-house, while the remaining three cohorts were retrieved from previously published literature (12–14).

Regarding the Tianjin cohort, the study enrolled a total of 49 participants between October 2021 and March 2022 at Tianjin First Central Hospital, including 34 individuals diagnosed with non-valvular atrial fibrillation and 15 healthy controls. Inclusion criteria for patients involved non-valvular AF, while exclusion criteria encompassed individuals with heart functional impairment, heart failure, structural heart disease, allergic conditions, inflammatory bowel disease, irritable bowel syndrome, autoimmune liver diseases, renal dysfunction, or tumors. Additionally, participants who had taken antibiotics or probiotics within the past month were also excluded.

Participants collected fresh fecal samples into sterile fecal collection tubes, which were promptly transferred to a −80°C freezer for subsequent experimental investigations. Blood plasma from each participant was collected using EDTA anticoagulant tubes, centrifuged at 3,000 rpm at room temperature for 10 min, and the supernatant plasma was carefully extracted and aliquoted into centrifuge tubes, then stored in a −80°C freezer for later experiments.

Data for the remaining three cohorts were retrieved from previously published public data sets. Subjects in these external cohorts were recruited based on the specific inclusion and exclusion criteria defined in the original studies (all comprising both AF patients and healthy controls), with approvals obtained from their respective institutional ethics committees.

### Bacteria strain, culture conditions, and freeze-dried powder

The *Lacticaseibacillus rhamnosus* J-5-3 and *Lactiplantibacillus plantarum* P-4 (Fig. S5A) used in this study were isolated from sauerkraut by the Tianjin Key Laboratory for Industrial Biological Systems and Bioprocessing Engineering (In this study, “*Lactobacillus”* is used as a broad term for the genus in the context of 16S rDNA sequencing analyses, while the updated genus names are used when referring to the specific intervention strains based on current taxonomic classification), Tianjin Institute of Industrial Biotechnology. These strains were selected specifically for their excellent acid tolerance and established safety profile for consumption. The bacteria were cultured in de Man, Rogosa, and Sharpe (MRS) broth under microaerophilic conditions at 37°C. The detailed composition of the MRS medium is provided in Table S6. These strains have been deposited at the China General Microbiological Culture Collection Center (CGMCC) and are associated with patent number CN115466702A. During the preparation of lyophilized powder, *L. rhamnosus* and *L. plantarum* strains were cultured at 37°C for 24 h. Trehalose was sterilized separately. The bacterial culture was centrifuged at 4°C at 5,000 rpm for 20 min, and the collected pellet was mixed with the lyophilization protective agent at a ratio of 1:2 (mass to volume). The composition of the protective agent was as follows: 11 g of skim milk, 3.03 g of trehalose, 9.50 g of glycerol, 3.5 g of sodium glutamate, and 100 mL of deionized water. This mixture was autoclaved at 115°C for 30 min and stored at 4°C. The mixture was pre-frozen at −80°C for 1 h, then freeze-dried for 48 h, and stored at 4°C. Before use in the gavage experiment, the lyophilized powder was rehydrated in sterile water at a ratio of 1:2 and diluted until the bacterial concentration reached 5 × 10⁹ CFU/mL.

### Animal model and treatments

Standard diet (SD) rats were purchased from Sipeifu (Beijing) Biotechnology Co., Ltd., with license number SCXK (Beijing) 2019-0010. The animals were housed at the Institute of Radiology Medicine, Chinese Academy of Medical Sciences, under specific pathogen-free conditions with a controlled environment at a temperature of 22°C ± 2°C and a 12-h light-dark cycle. Throughout the experimental procedures, all rats had unrestricted access to drinking water.

In *Lactobacillus* gavage experiments, after adaptively feeding for a week, rats were randomly divided into CK group (control, no treatment [*n* = 6]), AF group (disease model group [*n* = 9]), and PRO group (probiotic treatment group [*n* = 8]) according to body weight. The rats in the AF group and PRO group received a tail vein injection of a mixture of acetylcholine and calcium chloride at a dose of 1 mL/kg (CaCl_2_ at 10 mg/mL and acetylcholine at 66 μg/mL), once daily, whereas those in the CK group received a tail vein injection of saline as a sham treatment. After 10 days, subsequent injections were administered every other day via the tail vein. In the PRO group, rats were gavaged 1 mL of probiotic freeze-dried powder suspended in sterile water in the afternoon (probiotic concentration of 5 × 10^9^ CFU/mL, *L. rhamnosus* to *L. plantarum* ratio of 1:1). The other two groups were given an equivalent volume of sterile water, and this gastric gavage was continued for a total of 4 weeks. During this period, the rats’ body weight and mortality were monitored and recorded. After 4 weeks, the rats were anesthetized with isoflurane for subsequent testing and dissection experiments.

In antibiotic validation experiments, after adaptively feeding for a week, rats were randomly divided into PRO group (probiotic treatment group [*n* = 6]), ABX group (antibiotic treatment group [*n* = 8]), and PRO + ABX group (antibiotic and probiotic combination treatment group [*n* = 9]) according to body weight. All three groups were subjected to a daily tail vein injection of a mixture of calcium chloride and acetylcholine at a dose of 1 mL/kg (CaCl_2_ at 10 mg/mL and acetylcholine at 66 μg/mL). During the gastric gavage period, tail vein injections were administered every other day. In the PRO group, rats were orally administered 1 mL of probiotic freeze-dried powder dissolved in sterile water daily in the afternoon (concentration of 5 × 10^9^ CFU/mL, *L. rhamnosus* to *L. plantarum* ratio of 1:1). The ABX group received a gastric gavage of a mixture of antibiotics every other day in the morning (30 mg/kg vancomycin, 60 mg/kg neomycin, 60 mg/kg metronidazole, and 60 mg/kg ampicillin). The PRO + ABX group received both probiotics and antibiotics via gastric gavage (with a time interval of more than 6 h between antibiotic and probiotic administrations). This >6 h interval was specifically designed based on the rapid pharmacokinetic clearance in rodents and standard clinical guidelines (which typically recommend >2 h) to minimize direct spatiotemporal overlap between the antibiotic cocktail and the probiotics (17–20). Combined with the high inoculum size (5 × 10⁹ CFU/mL) and the intrinsic resistance of certain *Lactobacillus* species to specific antibiotics, this spaced regimen aimed to prevent the complete eradication of the administered probiotics, ensuring that they could reach the gut and allowing us to evaluate their interaction with the ABX-disrupted native microbiota. The feeding regimen was continued for 4 weeks, during which the rats’ body weight and mortality were regularly measured and recorded. After 4 weeks, the rats were anesthetized with isoflurane for subsequent testing and dissection experiments.

### Fecal DNA extraction

A Magnetic Soil and Stool DNA Kit (DP712-01, Tiangen, China) was used to extract total DNA from human and rat fecal samples. The extracted total DNA was subsequently amplified through PCR and sent to Shanghai Majorbio Bio-pharm Technology Co., Ltd. (Shanghai, China) for sequencing. Panbacterial 16S rRNA primers for 16S rRNA sequencing were used for the PCR amplification process (Table S9). DNA was amplified under the following reaction conditions: preincubate at 95°C for 3 min, followed by 30 cycles of denaturing at 95°C for 30 s, annealing at 55°C for 30 s, and extension at 72°C for 45 s.

### 16S rRNA gene sequencing and taxonomic annotation

For the Tianjin cohort, amplified DNA was sent to Shanghai Majorbio Bio-pharm Technology Co., Ltd. (Shanghai, China) for sequencing and subsequent quality control. Primers for sequencing and integrating data were listed in Table S3.

Raw sequence data for the remaining three cohorts were retrieved from the NCBI Sequence Read Archive (SRA) under the following accession numbers: Jiangxi (PRJNA806986), Shenyang (PRJNA832570), and Guangzhou (PRJNA785409). Detailed information regarding the sequencing target regions, primers, platforms, and sequencing depths for all four cohorts is summarized in Table S3. Sequencing data from all cohorts were processed using the DADA2 pipeline within the QIIME2 environment. Specifically, distinct import protocols were employed based on data format: single-end reads were processed using the SE import module, while paired-end reads utilized the PE import module. Subsequently, reads from both formats were trimmed to remove their respective primer sequences using the built-in truncation parameters in DADA2 and denoised based on their quality profiles. Taxonomic classification of amplicon sequence variants (ASV) was accomplished using the naive Bayes consensus taxonomy classifier for SILVA 138/16s_bacteria in QIIME2. Finally, microbial abundance data from the four sub-cohorts were integrated based on taxonomic annotations at the species level. To minimize technical variation across studies, batch effects were corrected using the MMUPHin method.

### Rarefaction analysis, α-diversity, β-diversity, and function prediction

The α-diversity analysis was performed on the Majorbio Cloud Platform (http://cloud.majorbio.com), and the rarefaction analysis and β-diversity assessment were conducted utilizing the “vegan” package in R version 4.3.1 (Table S11) to evaluate the feature richness in both the human cohort and the animal experiment.

### Machine learning approaches

Random forest (RF) classification was performed using the caret and randomForest packages in R. To ensure robust model construction and unbiased evaluation, all preprocessing and feature selection procedures were conducted in a strictly training-dependent manner. Specifically, within each modeling iteration, low-variance features were removed from the training set (variance <1e−5). Relative abundance profiles were then transformed using centered log-ratio (CLR) normalization to address data compositionality. The identical feature set retained after training-based filtering was subsequently applied to the corresponding test set.

Based on this standardized framework, two complementary validation strategies were implemented. First, samples underwent a stratified random split to maintain class balance, dividing them into a training set (70%) and an independent test set (30%). All model tuning and feature selection steps were performed exclusively within the training set. RF hyperparameters were optimized using 10-fold cross-validation. Feature selection was further evaluated using five repetitions of 10-fold cross-validation to estimate classification error as a function of the number of top-ranked features, where feature ranking was determined by mean decrease Gini importance. A reduced RF model using the selected feature set was retrained on the full training cohort and subsequently evaluated on the independent test set. Test samples were strictly excluded from feature ranking, parameter tuning, and model optimization to minimize overfitting.

Second, to rigorously assess cross-cohort generalizability, a leave-one-data set-out (LODO) cross-validation strategy was applied. In each LODO iteration, one entire independent data set was held out as the test cohort, while the RF classifier was trained on the remaining merged data sets using the same training-restricted preprocessing workflow described above. In LODO validation, RF hyperparameters were fixed (ntree = 500, with default mtry) to avoid overfitting. Finally, to identify consistently informative biomarkers, feature importance was calculated in each LODO iteration and summarized across all iterations by averaging mean decrease Gini values; the top 30 important biomarkers were extracted and compared with those identified in the primary feature selection procedure.

### Species rarity division and network visualization

In classifying species by rarity, species with relative abundances below 0.01% in all samples were identified as rare species, whereas species with relative abundances higher than 0.1% are identified as abundant species, and the rest were assigned to common species.

For rats experiments data, we primarily focused on the overall state of the microbial community; thus, we selected ASVs with a relative abundance sum >0.01% to construct the co-occurrence network. We specifically focused on edges exhibiting highly significant correlations (*P* < 0.01), signifying a robust co-occurrence pattern within communities. This suggests a higher likelihood of interactions among these taxa within the intestinal environment. To minimize false positives, we used CoNet for network inference (21). Pairwise associations among ASVs were assessed using Pearson, Spearman, Bray-Curtis, and Kullback-Leibler correlation methods simultaneously. Simultaneously, the optimal correlation threshold was determined based on random matrix theory (RMT) to ensure objective cutoff selection. Initial edge numbers were set at 1,000. For each edge and association measure, 1,000 permutation scores and bootstrap scores were calculated. To address compositional bias, taxon pairs were shuffled and renormalized before computing correlation measures. Edge- and measure-specific *P*-values were determined based on bootstrap distributions constrained by permutation means. Measure-specific *P*-values were combined using Brown’s method and adjusted for multiple testing with the Benjamini-Hochberg procedure. Edges supported by at least two correlation methods were retained, while those outside the 95% CI or with adjusted *P*-values >0.01 were removed. The final network was reconstructed from permutation and bootstrap data. The interactive platform “Gephi” and “Cytoscape” (default parameters set) were used to visualize module networks and calculate network parameters. Network robustness, natural connectivity, and vulnerability all refer to the methods of previous studies (22).

### Stochastic processes and niche breadth analysis

To evaluate the potential importance of stochastic processes in community assembly, we employed the neutral community model (NCM) using R scripts provided at https://github.com/Weidong-Chen-Microbial-Ecology/Stochastic-assembly-of-river-microeukaryotes, following the protocol described by Chen et al. (23). Furthermore, the normalized stochasticity ratio (NST) was utilized to quantify the relative contribution of deterministic versus stochastic processes. An NST value of 50% served as the boundary threshold, with values <50% indicating a dominance of deterministic assembly and values >50% indicating a dominance of stochastic assembly.

Additionally, following the method of Ahmad et al. (24), Levins’ niche breadth index was calculated for the microbial communities using the *“*spaa*”* package in the R statistical environment.

### Metagenomic prediction

Functional gene profiles were inferred from 16S rRNA sequences using the Phylogenetic Investigation of Communities by Reconstruction of Unobserved States (PICRUSt) tool. PICRUSt predicts metagenomic content from marker gene survey data through ancestral state reconstruction of quality-filtered 16S sequences. Additionally, BugBase was employed to characterize the predicted metagenomes based on biologically relevant bacterial phenotypes. BugBase analysis was performed using default parameters on the filtered sequence data.

### Untargeted metabolomics

Untargeted metabolomics analysis was performed on fecal and plasma samples obtained from a randomized subset comprising six AF patients and six healthy individuals selected from our sequencing cohort. The baseline clinical characteristics between AF patients and healthy controls were comparable (detailed in Table S4). Plasma and fecal samples preserved at −80°C were transported with dry ice to Shanghai Majorbio Bio-pharm Technology Co., Ltd. (Shanghai, China) for untargeted metabolomic analysis, with precautions taken to prevent repeated freeze-thaw cycles. The analysis was conducted on the Majorbio Cloud Platform (http://cloud.majorbio.com). Untargeted metabolomic profiling was performed using a Thermo UHPLC-Q Exactive HF-X system coupled with an ACQUITY HSS T3 column (100 mm × 2.1 mm, 1.8 μm; Waters, USA). The mobile phases for positive electrospray ionization (ESI+) mode consisted of 0.1% formic acid in water (A) and acetonitrile (B), while those for negative mode (ESI−) were 6.5 mM ammonium bicarbonate in water (C) and 6.5 mM ammonium bicarbonate in 95% methanol (D). The flow rate was maintained at 0.40 mL/min at 40°C, with an injection volume of 2 μL.

Raw LC-MS/MS data were processed using Progenesis QI software (Waters Corporation) for peak picking, alignment, and normalization. Metabolite annotation was conducted by matching the acquired m/z and MS/MS fragmentation patterns against the Human Metabolome Database (HMDB), METLIN, and our in-house reference library. Differential metabolites were identified based on a combination of statistical significance (*P* < 0.05) and fold change, followed by KEGG pathway enrichment analysis to interpret the biological implications.

### Quantification of LPS, IL-11, and TGF-β

The concentrations of LPS in rat plasma samples were quantified following the protocol outlined in the ET ELISA Kit (Shanghai Enzyme-linked Biotechnology Co., Ltd., ml061109-1). The concentrations of LPS in rat fecal samples were quantified following the protocol outlined in the ET ELISA Kit (Shanghai Enzyme-linked Biotechnology Co., Ltd., ml003197V). The concentrations of TGF-β and IL-11 in rat plasma were measured according to the manual of the Rat Transforming Growth Factor β (TGF-β) ELISA Kit (Shanghai Enzyme-linked Biotechnology Co., Ltd., ml028632) and the Rat Interleukin-11 (IL-11) ELISA Kit (Shanghai Enzyme-linked Biotechnology Co., Ltd., ml002949).

### Histological analysis

After euthanizing the mice, the heart tissues were isolated, and parameters such as heart size and weight were recorded. The heart tissues were then dissected into left and right atria and left and right ventricles. Subsequently, they were separately fixed in a 4% formaldehyde solution and stored at −80°C for further analysis. The atrial tissues fixed for 24–48 h were dehydrated with a series of ethanol solutions, followed by paraffin embedding. The paraffin-embedded blocks were sectioned into slices approximately 3 μm thick. After deparaffinization, the slices were stained with hematoxylin and eosin. Post-staining, they were washed, and the bluing step was performed with a lithium carbonate saturated solution, followed by counterstaining with eosin and another round of washing. Finally, the slices were mounted with neutral resin for imaging using a slide scanner. For Masson staining, the tissue sections were immersed in the Masson staining solution, followed by rinsing to remove excess dye. Subsequently, the sections were mounted with neutral resin, and the stained slices were subjected to image acquisition using a slide scanner. Images were acquired under consistent parameters to ensure comparability. The acquired images were imported into Image-Pro Plus software, where color deconvolution was applied to identify the blue-stained collagen fiber regions. Finally, the percentage of the collagen-positive area relative to the total tissue area was calculated to quantify the extent of fibrosis.

### Real-time quantitative PCR (qPCR)

The 16S rRNA gene of bacteria was quantitatively analyzed by real-time PCR using the CFX96 optical real-time detection system. The method of extraction of fecal DNA was the same as above. DNA was amplified under the following reaction conditions: preincubate at 95°C for 10 min; 95°C 15 s, 60°C 1 min, 40 cycles, 30 s later, read the board. Real-time PCR was performed three times for each DNA sample. The quantification of relative gene expression was carried out using the 2^−ΔΔCt^ method.

Species-specific primers were designed based on target gene sequences obtained from the National Center for Biotechnology Information (NCBI) database, using Primer Premier 5 software (Premier, Canada). Candidate primer sequences were selected to target regions conserved within the target species/strain and divergent from closely related non-target species. Primer specificity was assessed *in silico* using BLAST against the NCBI nucleotide database, and potential secondary structures and self-dimerization were avoided during selection. Beyond *in silico* validation, the specificity of the primers was empirically verified through PCR amplification using genomic DNA from several closely related congeneric species as templates. The resulting PCR products were analyzed via agarose gel electrophoresis to confirm the presence of a single band of the expected size and the absence of any non-specific amplification (Fig. S5E). Furthermore, a melting curve analysis was performed at the end of each qPCR run. The consistent observation of a single, sharp melting peak for each target species confirmed the high specificity of the primers and the reliability of the quantification (Fig. S5F). The final primer sequences and basic information are provided in Table S9.

### Statistical analysis

Statistical significance analysis was conducted using a two-tailed unpaired Student’s *t*-test, following verification of variance homogeneity through an *F*-test, or by employing one-way analysis of variance (ANOVA) for comparisons involving more than two groups, followed by Bonferroni multiple comparison post-tests (performed with GraphPad software, San Diego, CA). In cases where the assumptions of parametric tests were not met, differences between two groups were assessed using the nonparametric Mann-Whitney test. The exact number of replicates used in each experiment (*n*) is reported in the respective legends. The R packages used for statistics and visualization in this study are shown in Table S11.

## RESULTS

### Gut microbiota dysbiosis in AF patients

**Fig 1:**
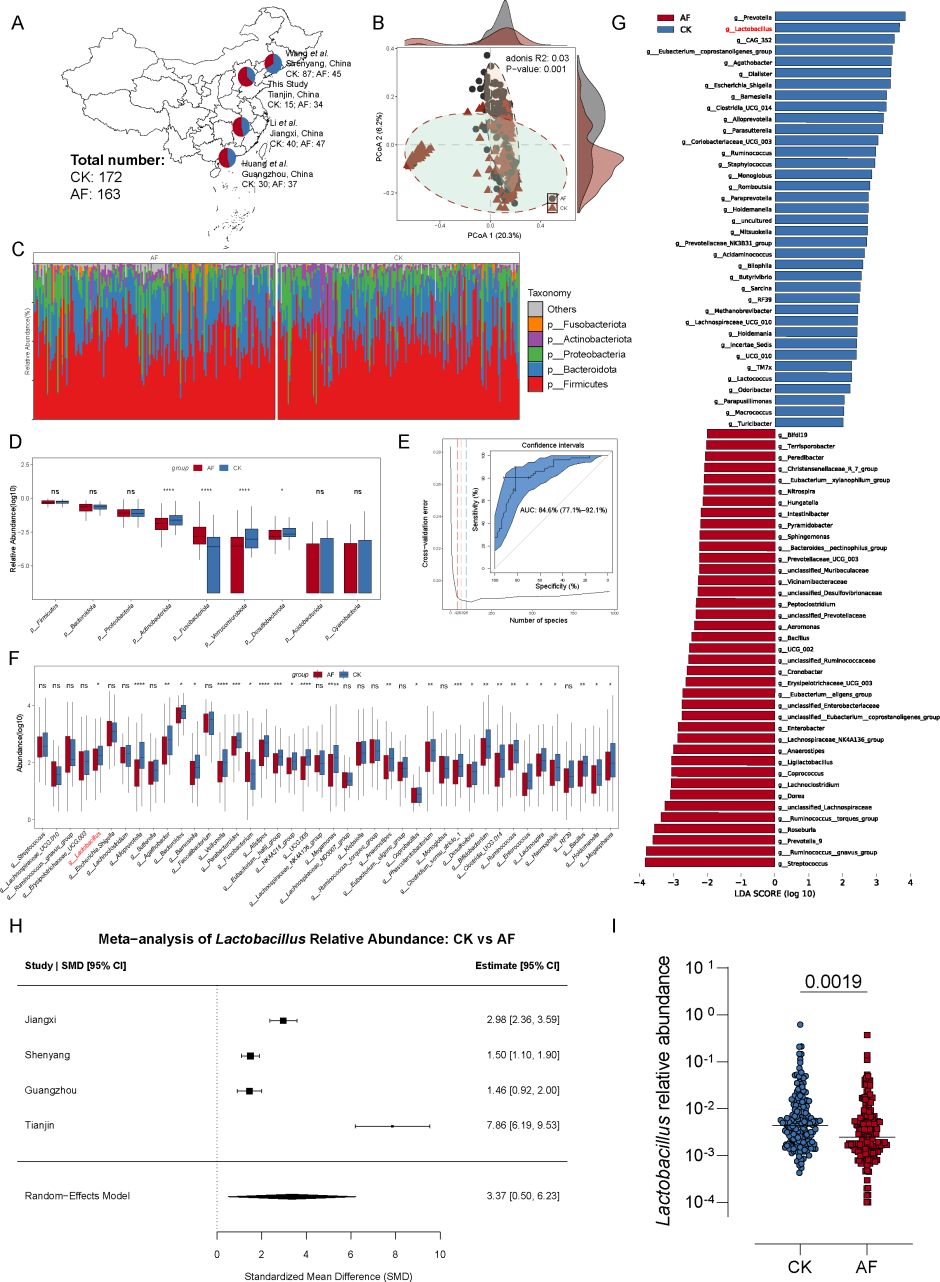
Gut microbiota dysbiosis in AF patients. (**A**) The China map represents a total of 335 samples from four cohorts. (**B**) β-diversity (principal coordinate analysis with Bray-Curtis dissimilarity) based on the species relative abundances and permutational multivariate analysis of variance (PERMANOVA) test. Red triangles represent samples from the AF group, while black circles represent samples from the CK group. The dashed lines indicate the 95% confidence intervals. (**C**) Abundance of bacterial taxa at the phylum level. Different colors represent different taxonomies. (**D**) Comparison of the relative abundances of phylum between the CK and AF groups (*P < 0.05, **P < 0.01, ***P < 0.001). (**E**) Five-repetition cross-validation screening of biomarkers and verifying the accuracy of the prediction of host disease status by a selected genus. (**F**) Comparison of the relative abundances of genus selected by random forest method (*P < 0.05, **P < 0.01, ***P < 0.001). (**G**) LefSE analysis screened differential species between CK and AF groups. (**H**) Meta-analysis of *Lactobacillus* relative abundance between CK and AF groups. (**I**) Comparison of the relative abundances of *Lactobacillus* between the CK and AF groups.

To elucidate the alterations in gut microbiota among patients with AF, we recruited 49 individuals from the Tianjin First Center Hospital and performed high-throughput 16S rRNA sequencing on stool samples. Besides, we also collected data from three other Chinese cohorts (Fig. 1A), which have been reported in previous studies, constructing an AF data set with a total of 335 samples (CK: 172, AF: 163). Among them, there were 132 samples in the Shenyang cohort (14) (PRJNA832570) after excluding two samples of all zeros. There were 67 samples in the Guangzhou cohort (13) (PRJNA785409) and 87 samples in the Jiangxi cohort (12) (PRJNA806986). Upon integrating data from the four cohorts, we observed an upward trend in the alpha diversity of the gut microbiota in AF patients compared to healthy controls, although this difference did not reach statistical significance (Fig. S1A). Furthermore, principal coordinate analysis (PCoA) was conducted on both the integrated data set and each individual sub-cohort. The results demonstrated discernible structural divergence between AF patients and healthy controls across the total population (Fig. 1B) and within each sub-cohort (Fig. S1B), indicating significant alterations in the gut microbiota composition associated with AF. At the phylum level, we found that *Actinobacteriota*, *Verrucomicrobiota,* and *Desulfobacterota* were enriched in healthy individuals, while *Fusobacteriota* was enriched in AF patients (Fig. 1C and D). At the genus level, *Fusobacterium* was significantly enriched in patients, suggesting a potential role in pathogenesis. Furthermore, using the random forest method (Fig. 1E and F), we screened 42 marker genera, including *Lactobacillus,* to predict the host status, and the area under the curve (AUC) index could reach 84.6% (95% CI: 77.1%–92.1%), indicating that the model prediction effect is good. In addition, leave-one-dataset-out (LODO) cross-validation further demonstrated the robustness of *Lactobacillus* as a biomarker (Fig. S1C and D). Subsequently, we used the LefSE method to cross-validate biomarkers screened by random forest (Fig. 1F and G). We found that *Lactobacillus* was significantly enriched in healthy individuals and decreased in patients, indicating that it has the potential to improve AF. To further validate the critical role of *Lactobacillus*, we compared its relative abundance between healthy controls and AF patients across the cohorts. We observed that *Lactobacillus* was depleted to varying degrees in AF patients in both the integrated data set and each individual sub-cohort (Fig. 1I; Fig. S2A through D and S3A). Furthermore, meta-analysis confirmed that this reduction pattern exhibited high consistency across all four cohorts (Fig. 1H; Fig. S3B). Collectively, these results underscore the pivotal role of *Lactobacillus* and suggest its potential as a therapeutic strategy for AF.

### Metabolic alterations and significant elevation of LPS in the feces and plasma of AF patients

**Fig 2:**
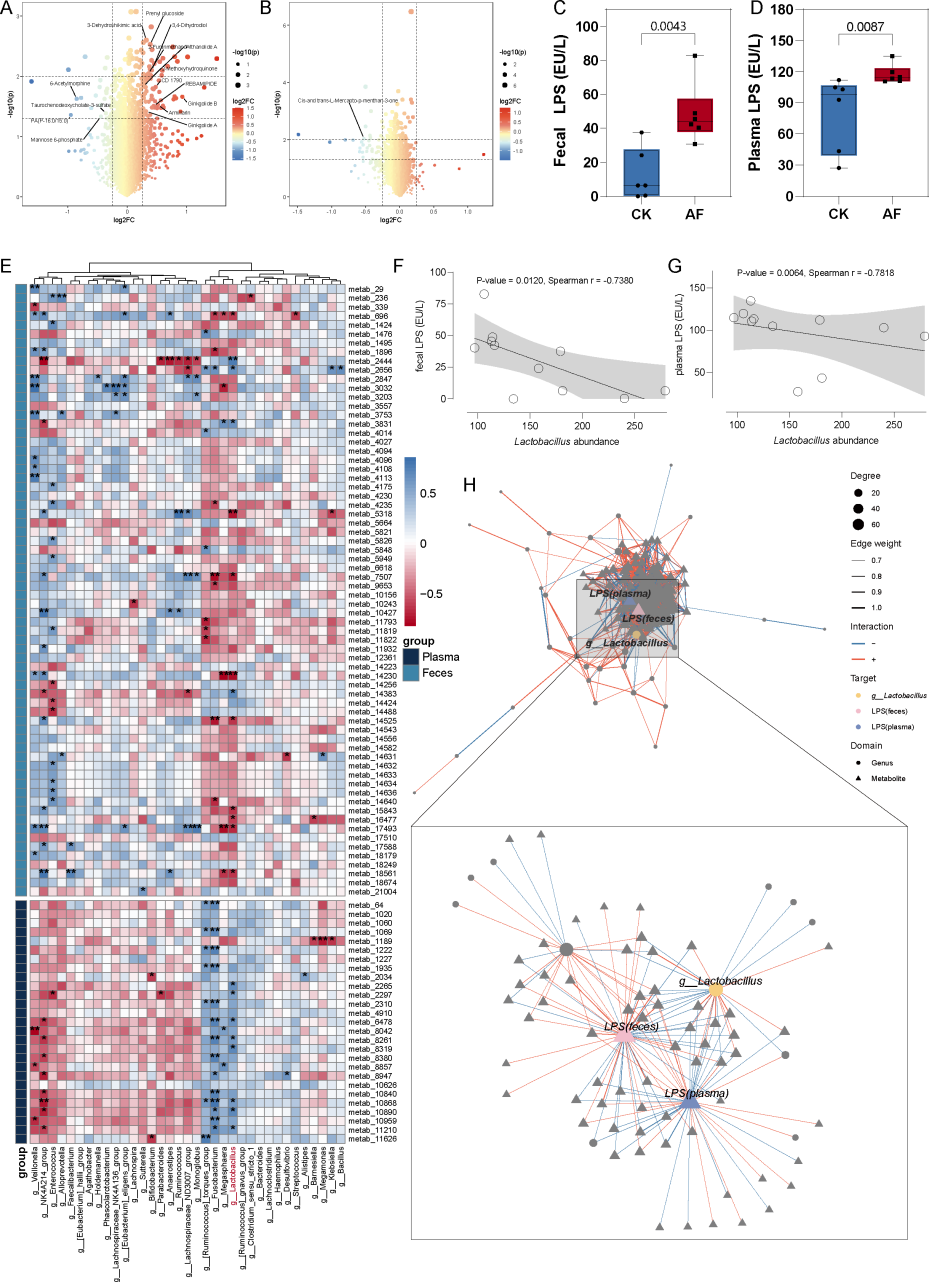
Metabolic alterations and significant elevation of LPS in the feces and plasma of AF patients. (**A and B**) Volcano plot of untargeted metabolomic data. Fold change indicates the changes in metabolites in the CK group compared with those in the AF group. *P*-value was calculated using the Student’s *t*-test. Spot color denotes the changing trend of metabolites and spot size denotes the significance. (**C and D**) Comparison of fecal and plasma LPS levels in the CK and AF groups. For fecal samples, *n* = 6 in the CK group, and *n* = 6 in the AF group. For plasma samples, *n* = 6 in the CK group, and *n* = 6 in the AF group. (**E**) Heatmap of Spearman correlations between the top 30 significantly differentially abundant genera and significantly differentially abundant metabolites (*P < 0.05, **P < 0.01, ***P < 0.001). (**F**) Correlation analysis of *Lactobacillus* abundance with LPS levels in the intestine and plasma. (**H**) Correlative network of 30 significantly differentially abundant genera and significantly differentially abundant metabolites.

To further elucidate the functional consequences of gut dysbiosis in AF, we performed untargeted metabolomics to profile the intestinal and systemic metabolic environments. In fecal samples, 68 metabolites exhibited significant differential abundance (Table S5), primarily spanning carbohydrate, energy, and amino acid metabolism. Specifically, microbiome-associated amino acid pathways, such as the biosynthesis of branched-chain amino acids (valine, leucine, and isoleucine) and phenylalanine metabolism, were significantly enriched in healthy individuals (Fig. S4A and C). Systemically, 27 metabolites were differentially abundant in the plasma of AF patients (Table S6). Pathways related to arginine, proline, glycine, and serine metabolism were characteristic of the healthy state, whereas the insulin resistance pathway was notably upregulated in AF patients (Fig. S4B and D). At the metabolite level, acetylcholine, an established pro-arrhythmic factor, was significantly elevated in AF plasma, while the cardioprotective metabolite gamma-glutamylglycine was depleted (Fig. 2A). Furthermore, integrative analysis demonstrated robust correlations between these key metabolites and the altered gut microbiota (Fig. 2E).

Given the potential inflammatory impact of endotoxemia on AF pathogenesis, we subsequently performed targeted quantification of LPS. ELISA analysis revealed a significant elevation in LPS levels within both the fecal samples and plasma of AF patients compared to healthy controls (Fig. 2C and D). Importantly, we found that the levels of LPS in the intestine and plasma were significantly negatively correlated with the abundance of *Lactobacillus* (Fig. 2F through H). This robust inverse relationship led us to hypothesize that the depletion of *Lactobacillus* may critically contribute to the pathological accumulation of LPS in AF, a mechanism we subsequently sought to validate in an *in vivo* model.

### *Lactobacillus* bacteria reduced LPS formation and improved AF

**Fig 3:**
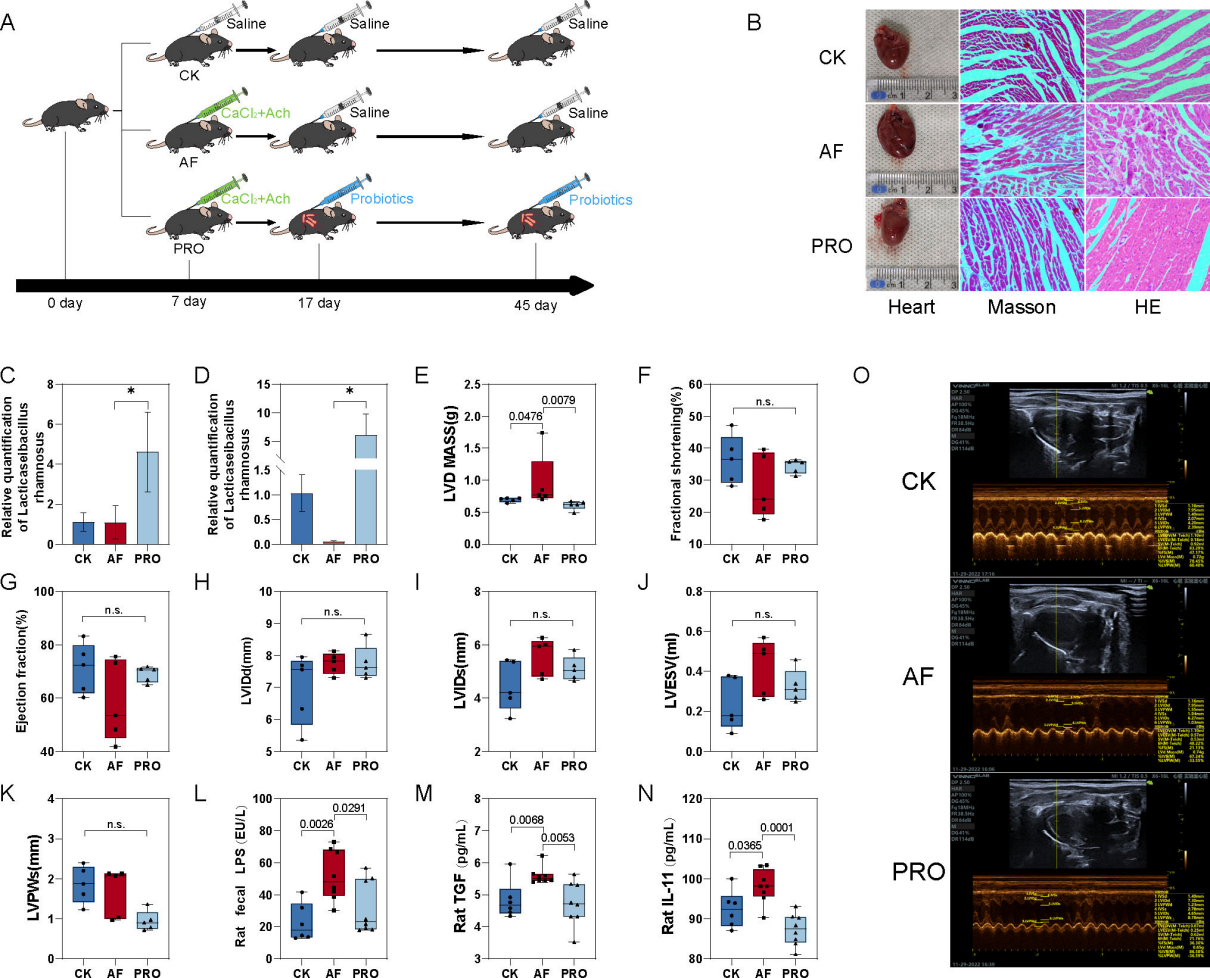
*Lactobacillus* bacteria reduced LPS formation and improved AF. (**A**) Schematic diagram of the experimental process of *Lactobacillus* improved AF (CK group [*n* = 6], AF group [*n* = 9], and PRO group [*n* = 8]). (**B**) Heart diagram (*n* = 6/group), Masson, and H&E staining. (**C and D**) Comparison of the relative abundance of *L. rhamnosus* and *L. plantarum* in stool samples from three groups. (**E–K**) LVd mass (**E**), fractional shortening (**F**), ejection fraction (**G**), LVIDd (**H**), LVESV (**J**), and LVPWs (**K**) were assessed by echocardiography in three groups (CK group [*n* = 5], AF group [*n* = 5], and PRO group [*n* = 5]). (**L**) Comparison of fecal LPS in CK group (*n* = 6), AF group (*n* = 8), and PRO group (*n* = 8). (**M and N**) Comparison of plasma TGF-α (**M**) and IL-11 (**N**) in CK group (*n* = 6), AF group (*n* = 8), and PRO group (*n* = 8). (**O**) Echocardiography in three groups (CK group, AF group, and PRO group).

*Lactobacillus* had been shown to alleviate various forms of cardiac injury (7, 25, 26), and our results indicated a significant reduction of *Lactobacillus* in AF patients (Fig. 1H and I; Fig. S3). To investigate the role of *Lactobacillus* in AF development, we selected two *Lactobacillus* strains, *L. rhamnosus* and *L. plantarum,* which have been used to protect against atherosclerosis (27) and coronary artery disease (28), and administered them to an AF rat model induced by acetylcholine and CaCl_2_ (Fig. 3A). Quantitative PCR (qPCR) confirmed successful colonization of *L. rhamnosus* and *L. plantarum* in the rats’ colons (Fig. 3C and D). Compared with AF rats, heart size in healthy rats was significantly smaller and *Lactobacillus* treatment improved this symptom (Fig. 3B). Histological analysis using hematoxylin and eosin (HE staining) and Masson staining revealed that AF model rats exhibited a more disordered cardiomyocyte arrangement, a more obvious interstitial collagen fiber deposition, and a greater fibrotic area ratio compared to healthy rats (Fig. 3B; Fig. S5C). Besides, the assessment showed that left ventricular (LVd) mass in the AF group was significantly higher than that in the CK and PRO groups, whereas other indices were not statistically significant (Fig. 3E through K and O; Fig. S6A through D). However, compared with the other groups, there were downregulated trends in the fractional shortening and ejection fraction of the AF group (Fig. 3F and G), and the indices (left ventricular diastolic dimension (LVIDd), left ventricular systolic diameter (LVIDs), left ventricular end-systolic volume (LVESV), and left ventricular posterior wall (LVPWs) trended to increase (Fig. 3H through K). Moreover, in the AF group, the electrocardiogram showed the disappearance of P-waves, while the other two groups exhibited no abnormalities (Fig. S6E). Those showed the symptoms in acetylcholine and CaCl_2_ combined treatment rats had been ameliorated in *Lactobacillus* combination treatment rats. All results above suggested that the *Lactobacillus* combination could improve AF.

To elucidate the mechanism underlying the beneficial effects of the *Lactobacillus* combination on AF, we used PICRUSt2 to predict gut microbiota functions in AF rats and *Lactobacillus* combination-treated rats, focusing on differentially enriched functional pathways. The results indicated that the gut microbiota in the PRO group was enriched in pathways related to bisphenol degradation, drug metabolism, retinol metabolism, and xenobiotic metabolism, whereas pathways associated with LPS biosynthesis, phenazine biosynthesis, mineral absorption, and D-arginine and D-ornithine metabolism were enriched in the AF group (Fig. S7N). Based on these findings, we focused on LPS, a well-established pro-inflammatory factor (29), as a target biomarker. Subsequent fecal analysis revealed that LPS concentrations were significantly higher in AF rats than in healthy controls, whereas *Lactobacillus* combination-treatment markedly reduced LPS levels (Fig. 3F).

IL-11 and TGF-β were closely related to cardiovascular fibrosis (30), which could be activated by LPS (31). Thus, we detected the IL-11 and TGF-β concentration in the serum to further investigate the mechanism of our *Lactobacillus* combination on improving AF. In the AF group, the serum concentrations of IL-11 and TGF-β were significantly higher than those in the CK group, whereas the *Lactobacillus* combination could significantly improve this phenomenon (Fig. 3G and H). Collectively, these results suggest that inflammation is a potential contributor to the pathogenesis of AF and that gut-derived LPS may play a key mediating role in this process. In addition, *L. rhamnosus* and *L. plantarum* were positively associated with the amelioration of AF biomarkers and the reduction of fecal and serum LPS.

### The target of *Lactobacillus* bacteria is the gut microbiota

**Fig 4:**
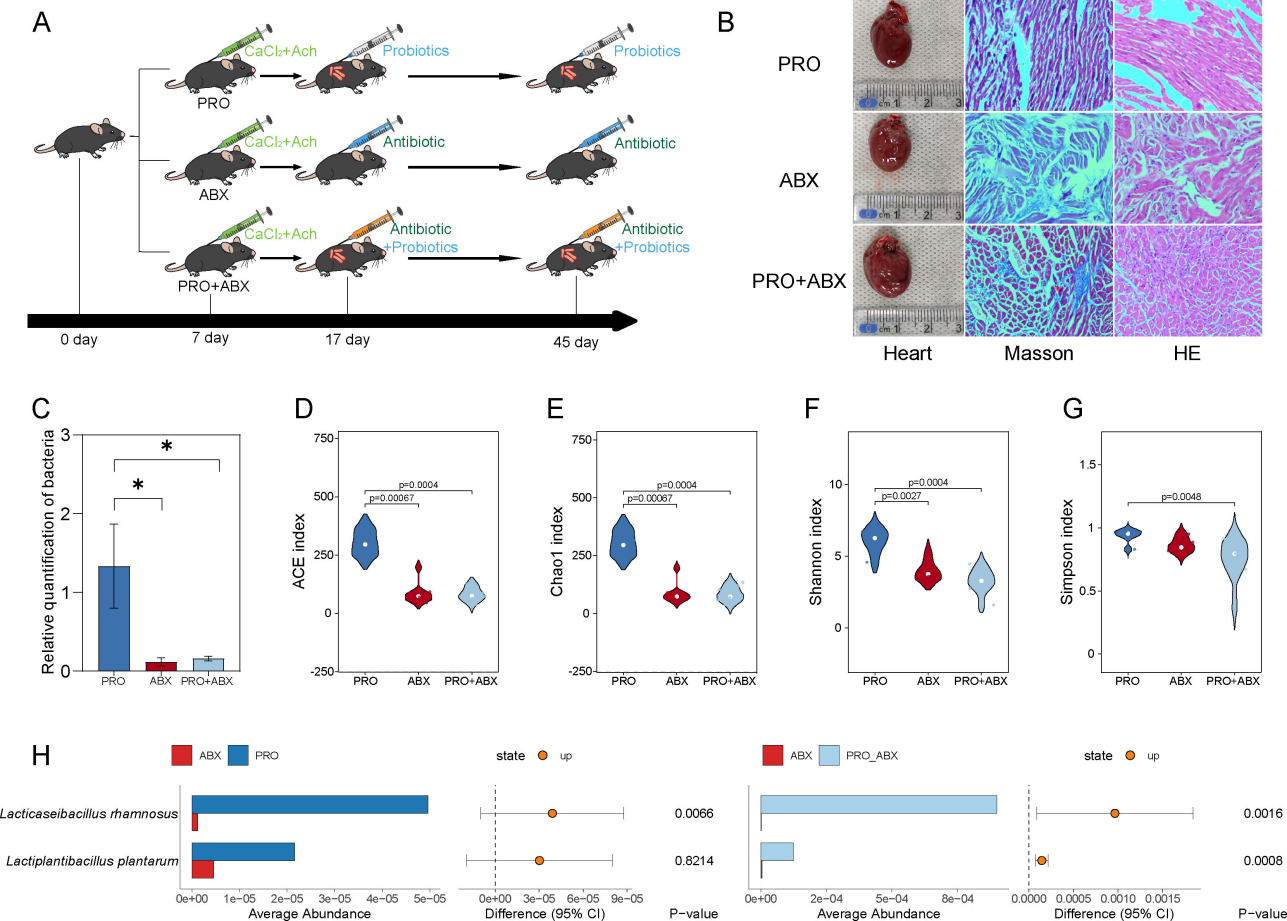
The target of *Lactobacillus* is the intestinal microbiota. (**A**) Schematic diagram of the experimental process of *Lactobacillus* affected on gut microbiota improved AF (PRO group [*n* = 6], ABX group [*n* = 8], and PRO + ABX group [*n* = 9]). (**B**) Heart diagram (PRO group [*n* = 3], ABX group [*n* = 4], and PRO + ABX group [*n* = 4]), Masson, and H&E staining. (**C**) Comparison of the relative abundance of bacteria in stool samples from three groups. (**D–G**) α-diversity ACE index (**D**), Chao1 index (**E**), Shannon index (**F**), and Simpson index (**G**) based on the species relative abundances in the PRO (*n* = 6), ABX (*n* = 8), and PRO + ABX (*n* = 9) groups. Two-tailed Wilcoxon rank-sum test was used to determine intergroup significance. (**H**) Comparison of the relative abundance of *L. rhamnosus* and *L. plantarum* in the PRO, ABX, and PRO + ABX groups. The left panel shows the mean relative abundance, while the right panel illustrates the 95% confidence intervals (CIs) for the difference in mean proportions. Statistical significance was determined using the Wilcoxon rank-sum test to compare differences between groups. *P*-value < 0.05 was considered statistically significant.

To further validate the impact of *Lactobacillus* bacteria on gut microbiota, we designed an experiment comprising three groups: *Lactobacillus* combination treatment (PRO group), antibiotic treatment (ABX group), and *Lactobacillus* combination with antibiotic treatment (PRO + ABX group) (Fig. 4A). The results from 16S rRNA sequencing indicated that the ACE, Chao1, and Shannon diversity indices in antibiotic treatment and *Lactobacillus* combination with antibiotic treatment were significantly lower than those in *Lactobacillus* combination treatment (Fig. 4D through G). The qPCR results revealed that the bacteria quantification in the *Lactobacillus* combination treatment was far higher than the other two treatments (Fig. 4C), and *L. rhamnosus* and *L. plantarum* exhibited high colonization in the PRO + ABX group (Fig. 4H). Furthermore, we constructed co-occurrence networks to evaluate the overall ecological shifts in the gut microbiota. The results revealed that the ecological networks in both the ABX and PRO + ABX groups had almost entirely collapsed. In contrast, the PRO group successfully retained specific network modules encompassing *Lactobacillus* (Fig. S5B). Furthermore, compared to the PRO group, heart size was significantly larger in the ABX and PRO + ABX groups. Histological evaluations using HE and Masson staining demonstrated that atrial tissue from the PRO group exhibited neatly arranged cells with reduced collagen fiber content and minimal interstitial collagen deposition. In contrast, samples from the ABX and PRO + ABX groups showed disorganized cardiomyocyte arrangement, with significantly increased areas of myocardial collagen deposition and fibrosis (Fig. 4B; Fig. S5C). These results underscore that the therapeutic target of our *Lactobacillus* combination is the gut microbiota and that probiotic consortia cannot function independently of the native microbial community.

### The impact of *Lactobacillus* on the colonization of rare species

**Fig 5:**
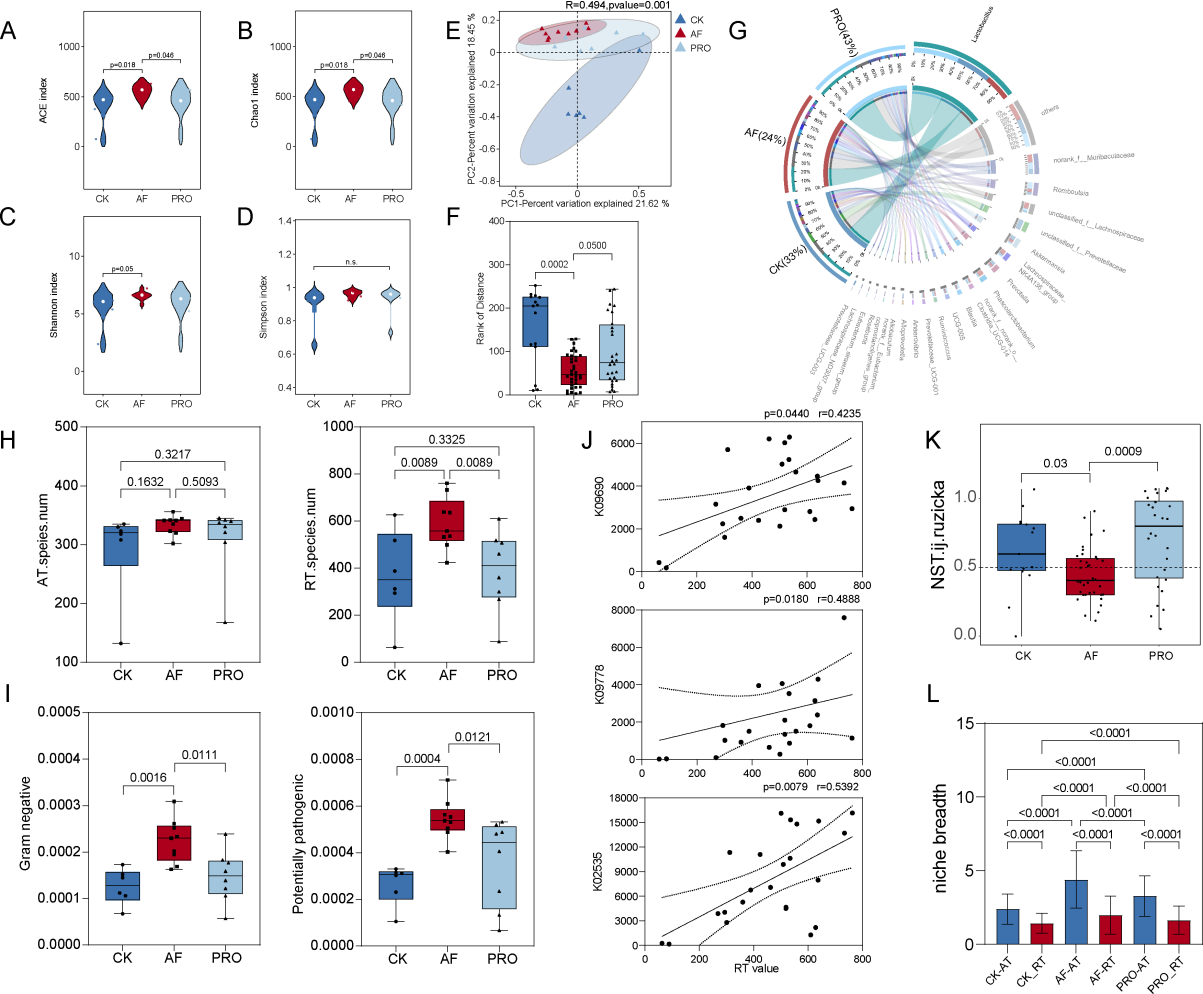
*Lactobacillus* bacteria reduced the production of rare species. (**A–D**) α-Diversity ACE index (**A**), Chao1 index (**B**), Shannon index (**C**), and Simpson index (**D**) based on the species relative abundances in the CK (*n* = 6), AF (*n* = 9), and PRO (*n* = 8) groups. Two-tailed Wilcoxon rank-sum test was used to determine intergroup significance. (**E**) β-Diversity (principal coordinate analysis with Bray-Curtis dissimilarity) based on the species relative abundances, permutational multivariate analysis of variance (PERMANOVA) test. Two-tailed Wilcoxon rank-sum test was used to determine intergroup significance. (**F**) Analysis of similarity (ANOSIM) showing the differences in microbial community structures among three groups (two-tailed Wilcoxon rank-sum test). (**G**) Circos diagram of distribution at the genus level. (**H**) The distribution of number of ASVs based on abundance. (**I**) The abundance of gram-negative (**M**) and potentially pathogenic (**N**) bacteria. (**J**) Spearman’s correlation between the ASVs number of RT and the LPS biosynthesis gene. Statistical significance was evaluated by two-tailed nonparametric Mann–Whitney test (**A–D, I–J**). (*P < 0.05, **P < 0.01, ***P < 0.001). (**K**) Three groups of NST scores based on the normalized stochasticity ratio model. (**L**) Niche breadths of rare and abundant species in the three groups.

To further investigate the effects of *Lactobacillus*, we employed 16S rRNA sequencing to analyze changes in gut microbiota among three experimental groups. Rank-abundance and rarefaction curves indicated that the sample size was adequate and the species distribution was uniform (Fig. S7A and B). The 16S rRNA sequencing results demonstrated that all the ACE, Chao1, Observed, and Shannon indices were significantly elevated in the gut microbiota of the AF group (Fig. 5A through D; Fig. S8A), consistent with findings from the human cohort (Fig. 1A and B). Notably, *Lactobacillus* treatment led to a gut microbiota structure of AF rats that was more similar to that of the CK group. Additionally, PCoA analysis revealed significant divergence among the microbial communities of the three groups (Fig. 5E and F). Furthermore, to preclude potential data bias arising from *Lactobacillus* abundance, we recalculated the microbial diversity after excluding this genus. The results remained consistent with the aforementioned findings: AF induced gut microbiota dysbiosis, whereas *Lactobacillus* treatment restored the microbial community structure (Fig. S8C and D). At the genus level, *Lactobacillus* represented the largest proportion within the overall communities, while the AF group exhibited the smallest proportion. Probiotic supplementation significantly increased the percentage of *Lactobacillus* within the gut microbiota (Fig. 5G and Fig. S3D).

In our study, we classified whole ASVs into three taxa, including rare taxa (RT), common taxa (CT), and abundant taxa (AT), according to the previous study (32), and we mainly discussed the role of rare and abundant species. Notably, there was no significant difference in the number of AT ASVs among the three groups (Fig. 5H). Compared with gut microbiota in the CK group, the RT ASVs number in the AF group was significantly higher, whereas *Lactobacillus* treatment significantly reduced their amounts (Fig. 5H). To verify the robustness of our findings, we recalculated the number of RT ASVs after excluding *Lactobacillus*, and the results remained consistent with the initial analysis (Fig. S8B). Meanwhile, PICRUSt2 analysis indicated that the ASVs number of RT was significantly correlated with several genes encoding proteins involved in LPS synthesis, assembling, and transporting proteins such as K09690 (LPS transport system permease protein), K09778 (Kdo2-lipid IVA 3′ secondary acyltransferase), and K02535 (UDP-3-O-[3-hydroxymyristoyl] N-acetylglucosamine deacetylase) (Fig. 5J). Using BugBase to predict organism-level microbiota phenotype (33), we observed that the abundance of potentially pathogenic and gram-negative bacteria significantly increased in the RT of AF group, while *Lactobacillus* treatment attenuated this increasing trend (Fig. 5I). Furthermore, the abundance of gram-negative bacteria in the RT exhibited a significant positive correlation with genes encoding proteins involved in LPS synthesis, assembling, and transporting proteins including K09774 (LPS export system protein LptA), K11720 (LPS export system permease protein), K06861 (LPS export system ATP-binding protein), K07271 (LPS cholinephosphotransferase), K00677 (UDP-N-acetylglucosamine acyltransferase), K00979 (3-deoxy-manno-octulosonate cytidylyltransferase), K01627 (2-dehydro-3-deoxyphosphooctonate aldolase), K02535 (UDP-3-O-[3-hydroxymyristoyl] N-acetylglucosamine deacetylase), and K02536 (UDP-3-O-[3-hydroxymyristoyl] glucosamine N-acyltransferase) (Fig. S3E through M).

Besides, using the normalized stochasticity ratio (NST) model (34), we found NST in CK and PRO groups were both higher than 0.5, indicating that the microbial communities in these groups were primarily assembled through stochastic processes. Conversely, the NST value in the AF group was lower than 0.5, suggesting that the microbiota in this group was predominantly assembled through deterministic processes (Fig. 5K). Furthermore, we calculated the niche widths of rare and abundant species across the three communities. The results demonstrated that the niche breadth for both abundant and rare taxa in the AF group was greater than that observed in the other groups, with abundant taxa consistently exhibiting broader niche widths than rare taxa across all groups (Fig. 5L).

### *Lactobacillus* bacteria restore the stability of the intestinal microbiota network

**Fig 6:**
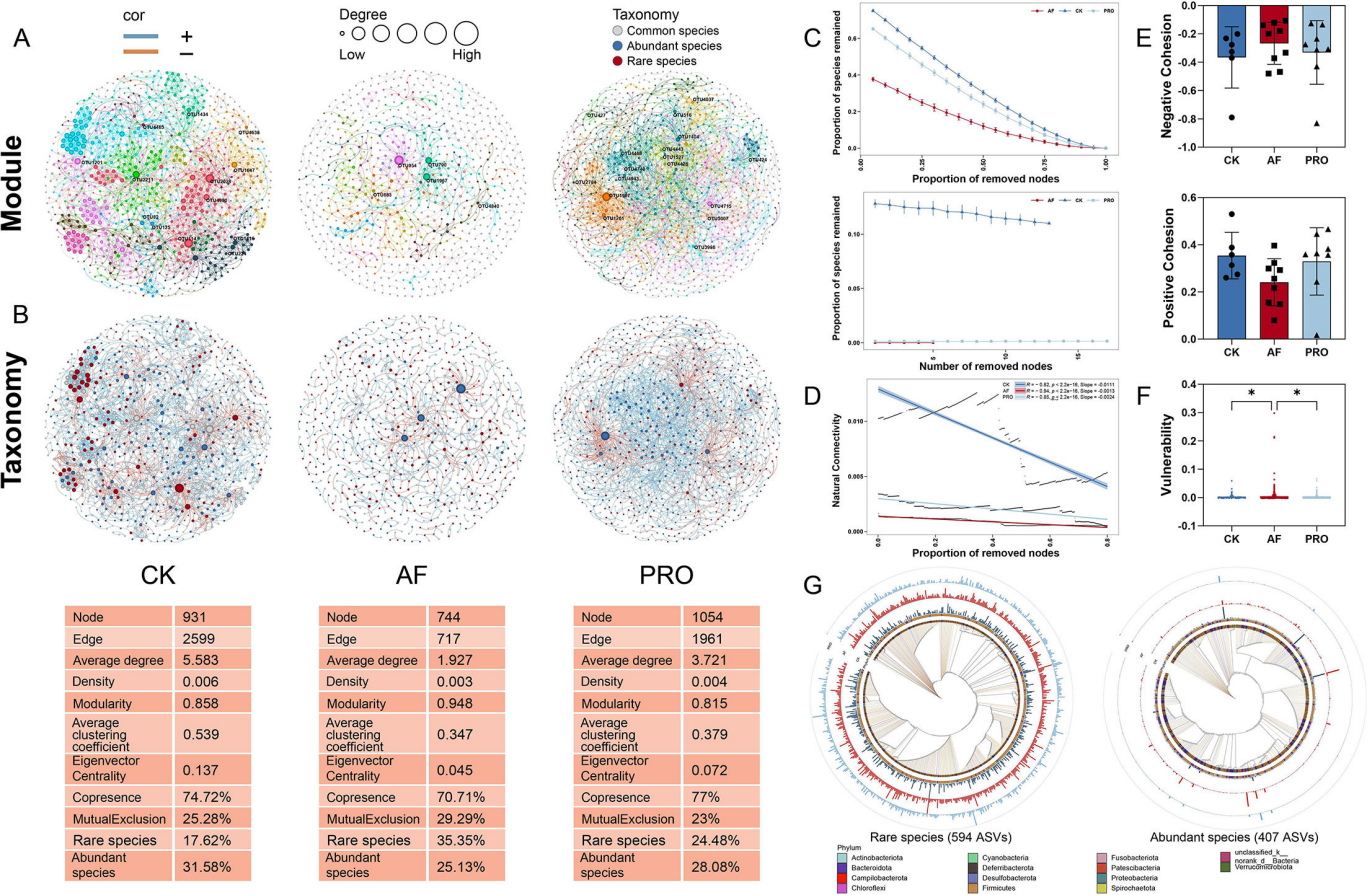
*Lactobacillus* bacteria restore the stability of the intestinal microbiota network. (**A and B**) Co-occurrence networks based on CoNet network inference. Node size was proportional to its degree. Node color indicated different modules (**A**) and taxonomy (**B**). Edges of networks derived from different modules (**A**) and correlation (**B**). (**C**) The robustness of the remaining nodes is calculated by randomly removing nodes and hubs. (**D**) The natural connectivity of the remaining nodes is calculated by randomly removing nodes. (**E**) Negative and positive cohesion of networks in three groups. (**F**) Vulnerability of networks in three groups. (**G**) Phylogenetic trees of rare and abundant species that make up the network. Different colors of branches represent different phyla.

To further illuminate the role of *Lactobacillus* bacteria in restructuring gut microbiota, we constructed network models based on the interaction relationships among the three treatment groups. The networks revealed that the gut microbiota in the CK group exhibited the highest number of nodes and interactions, which were significantly reduced in the AF group (Fig. 6A and B). Notably, *Lactobacillus* treatment markedly restored the number of nodes and edges, indicating that the interactions between strains in the gut microbiota of the CK and PRO groups were stronger than those observed in the AF group. Consequently, the networks of the CK and PRO groups were more complex. Further analysis showed that the average network degrees for the CK, AF, and PRO groups were 5.583, 1.927, and 3.721, respectively. The average clustering coefficients were 0.539, 0.347, and 0.379, respectively. The network densities of the CK, AF, and PRO were 0.006, 0.003, and 0.004, respectively. These results collectively indicate that the complexity of the microbial network was significantly diminished due to AF, whereas *Lactobacillus* treatment effectively restored the associations between microorganisms, enhancing network complexity.

Moreover, the modules identified through ZiPi analysis (35), which included module hubs, were considered principal modules and were distinguished by different colors in our network (Fig. 6A; Fig. S9). We observed distinct modules in CK and PRO networks, comprising 9 and 10 main modules, respectively. In contrast, the AF network contained only four main modules. In addition, the eigenvector centrality of the CK, AF, and PRO were 0.137, 0.045, and 0.072, respectively. This indicates that bacteria in the CK and PRO networks clustered and interacted with other members more frequently compared to the AF group. Interestingly, the modularity of the AF network was 0.948, which was higher than that of the CK and PRO groups, which exhibited modularity values of 0.858 and 0.815, respectively. This suggests that microorganisms in the AF network had a preference for forming smaller modules. In summary, all parameters collectively indicate that *Lactobacillus* treatment effectively reshaped the complexity of gut microbiota in AF rats.

To assess network robustness among the three groups, we randomly removed 5% of the total nodes and module hubs and recalculated the robustness iteratively until all nodes and hubs were removed. The results revealed that gut microbiota in CK group possessed the highest robustness, while that of AF rats demonstrated significant instability. In contrast, *Lactobacillus* combination treatment made gut microbiota regain robustness (Fig. 6C). In addition, we evaluated the resistance of microbial networks to disturbances by selectively removing network nodes that altered the amplitude of natural connectivity. The findings revealed that the resistance to disturbances in the CK and PRO groups was notably more stable compared to the AF group (Fig. 6D).

Cohesion is the measurement that was used to quantify network connectivity (36). The networks possessing high positive and low negative cohesions showed higher robustness and persistence (22). Subsequently, we compared cohesion among three networks, which indicated that networks in CK and PRO groups possessed higher positive and lower negative cohesions than that in AF group (Fig. 6E). Interestingly, we also found that the cohesion of the network was significantly negatively correlated with LPS levels (Fig. S10), which might suggest that the stability of the network helps reduce the release of LPS by the gut microbiota. In addition, the vulnerability that quantifies the node ability on network efficiency in AF groups (22) was higher than that in the other two groups, which suggested that the network in AF group was more likely to be damaged in the case of nodes loss (Fig. 6F). All results above mentioned suggested that AF reduced network complexity and destabilized community stability, whereas *Lactobacillus* restored the community to a state closer to that of a community of healthy individuals.

### *Lactobacillus* bacteria reshape the “LPS-reservoir modules” of the intestinal microbiota network

**Fig 7:**
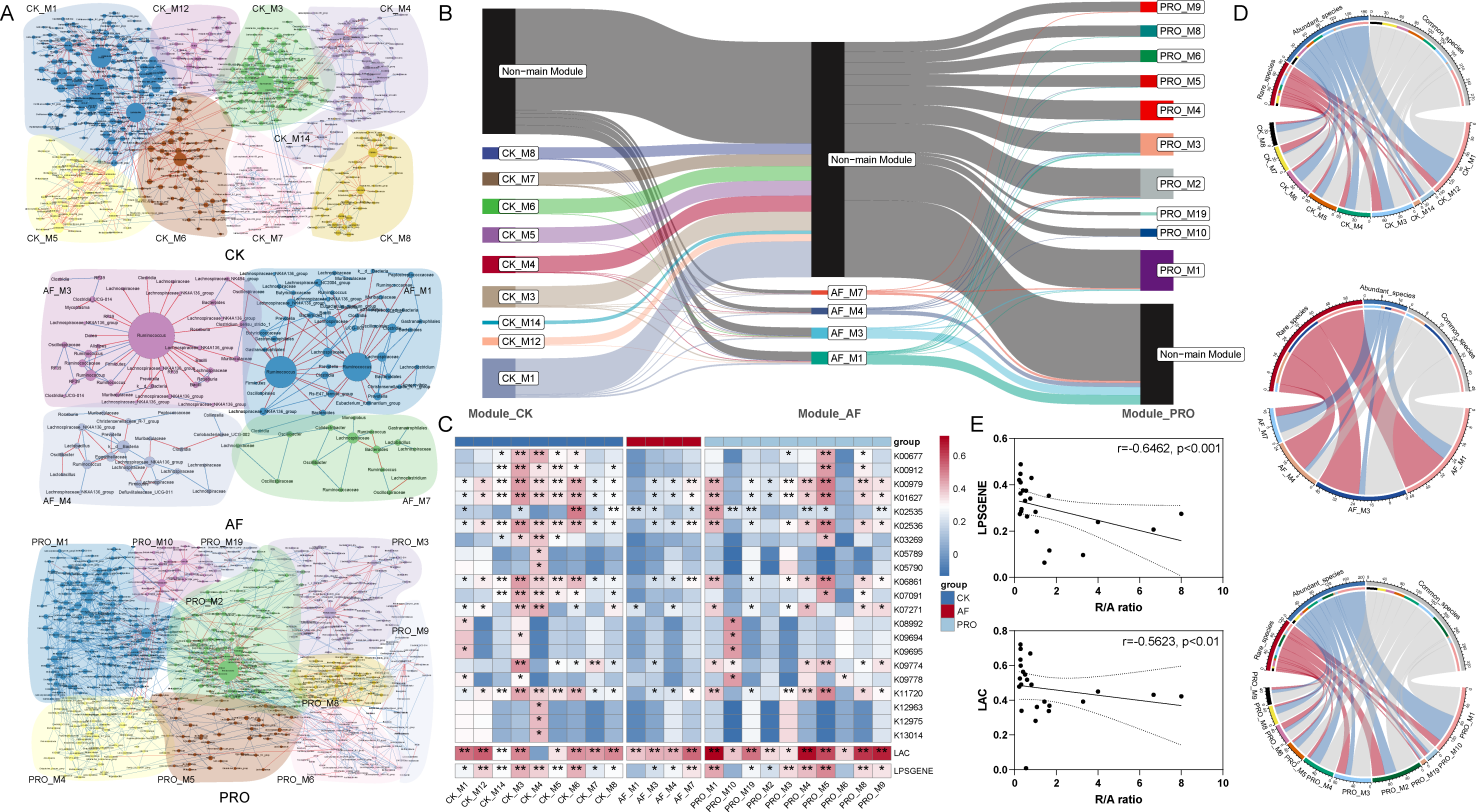
*Lactobacillus* bacteria reshape the “LPS-reservoir modules” of the intestinal microbiota network. (**A**) Co-occurrence networks derived from the main modules of networks in three groups. Nodes are sized by degrees and colored by modules. Labels of nodes are the genus level of ASVs. Edges with red represent negative correlative and edges in blue represent positive correlation. (**B**) Alluvial plot showing the shuffling of species between community modules identified in the CK (left), AF (middle), and PRO (right). Different widths of links represent different numbers of migrated ASVs. (**C**) Heatmap of Spearman correlations between the main modules and LPS synthetic genes as well as *Lactobacillus* abundance (*P < 0.05, **P < 0.01, ***P < 0.001). (**D**) Circos diagram of the distribution of abundant as well as rare taxa in the main modules. (**E**) Spearman’s correlation between R/A (rare taxa to abundant taxa) ratio and the correlation value between module composition and LPS synthetic genes as well as *Lactobacillus* abundance.

To further decipher the association between microbial community stability and the abnormal accumulation of LPS, we conducted an in-depth analysis of the composition and functionality of the main modules. Our findings revealed two and one modules with *Lactobacillus* as the module hub in the networks of the CK and PRO groups, respectively, whereas no such modules were identified in the AF group (Fig. 7A). Notably, we observed that nearly all main modules were significantly correlated with the abundance of *Lactobacillus* (Fig. 7C). Additionally, many of these main modules exhibited significant associations with genes involved in LPS synthesis, with the correlations in the CK and PRO groups being more pronounced than those in the AF group (Fig. 7C). Given that modules serve as stable components within the network (37), it appears that the main modules function as “LPS-reservoir modules,” facilitating the storage of strains capable of synthesizing LPS, thereby mitigating its release during species extinction. However, the size and number of these “LPS-reservoir modules” in the AF group were considerably diminished compared to those in the CK group, although *Lactobacillus* treatment facilitated their recovery. Furthermore, the composition of these “LPS-reservoir modules” exhibited considerable variation between the groups. A substantial number of species from the “LPS-reservoir modules” in the CK group were fragmented into smaller, non-main modules or free nodes in the AF group, while these species were recombined to form “LPS-reservoir modules” in the PRO group (Fig. 7B).

Additionally, we found that both abundant and rare species played crucial roles in forming “LPS-reservoir modules.” Specifically, 594 ASVs of rare species and 407 ASVs of abundant species contributed to network construction (Fig. 6G), but rare species were present in a smaller proportion within most “LPS-reservoir modules” compared to abundant species (Fig. 7D). Using the correlation between modules and LPS synthesis-related genes as an indicator of the “LPS-reservoir module” LPS storage capacity, we observed that this capacity diminished as the R/A ratio (ratio of rare species to abundant species) increased (Fig. 7E). This finding indicates that rare species contributed less to LPS storage capacity than abundant species in the “LPS-reservoir modules.” Furthermore, we used the correlation between modules and *Lactobacillus* abundance to assess *Lactobacillus*’s role in promoting "LPS-reservoir module" formation. Our results showed that the facilitative effect of *Lactobacillus* on "LPS-reservoir modules" decreased as the proportion of rare species increased, suggesting that the R/A ratio may be a critical factor through which *Lactobacillus* influences "LPS-Reservoir Modules" (Fig. 7E).

## DISCUSSION

Given its diverse roles in immune education, endocrine, and neurochemical signaling, and modulation of drug metabolism, the gut microbiota has garnered increasing interest as a therapeutic target for AF (5). However, limited research cohorts and a paucity of mechanistic studies have left the gut microbiota-AF relationship poorly understood. To address these gaps, we established the largest Chinese AF-related gut microbiota cohort to date, aiming to delineate intestinal ecological alterations in affected patients. Diversity analyses revealed marked alterations in AF patients’ gut microbiota. At the phylum level, *Fusobacteriota* was significantly enriched, whereas *Actinobacteriota* and *Verrucomicrobiota* were depleted. At the genus level, *Fusobacterium* increased markedly, while *Lactobacillus*, *Alloprevotella*, and *Alistipes* were significantly reduced. *Fusobacterium* has been previously identified as a pathogen associated with multiple diseases and serves as a major source of intestinal LPS, a finding that aligns with our results (38).

Furthermore, to investigate the gut microbiota-AF relationship, we focused on *Lactobacillus* due to its pronounced differential abundance between healthy controls and AF patients, as well as its established safety profile and ecological adaptability (7). Targeted metabolomics analysis revealed an inverse correlation between *Lactobacillus* abundance and LPS levels, which is consistent with the results of the *in vitro* colon model in previous studies (39). As a major structural component of the gram-negative bacterial outer membrane (40), LPS was shown to be highly relevant to the occurrence of AF (41). Daniele et al. measured serum LPS in 912 AF patients and linked elevated LPS to a higher AF risk (41). Zhang et al*.* used an FMT rat model to show that microbiota from young donors attenuated age-related AF, identifying LPS as a key mediator (4). Thus, we hypothesize that *Lactobacillus* mitigates AF by lowering LPS levels.

To test our hypothesis, we administered *Lactobacillus* orally in an AF rat model. *Lactobacillus* treatment ameliorated key disease features, including cardiac enlargement, myocardial fibrosis, and elevated plasma inflammatory markers, and significantly reduced intestinal LPS levels, confirming our hypothesis. An antibiotic depletion experiment further demonstrated that *Lactobacillus'*s protective effects were abolished in the absence of gut microbiota, indicating that *Lactobacillus*’s ability to lower intestinal LPS depends on the resident microbial community. However, as a cell-wall component of gram-negative bacteria, LPS is typically released only upon bacterial lysis or turnover (16). Therefore, we postulate that the aberrant LPS levels observed in the disease state may stem, in part, from the increased instability of the overall intestinal ecosystem. Subsequently, adopting an ecological perspective, we investigated how *Lactobacillus* modulates the gut microbial community to restrict LPS release and thereby ameliorate AF.

To elucidate how *Lactobacillus* modulates gut ecology to reduce LPS, we constructed molecular ecological networks to investigate the mechanism. Our findings indicate that gut microbiota networks in low-LPS groups (CK and PRO) are more complex than those in the high-LPS group (AF). The AF group exhibited fewer network nodes and connections compared to healthy controls, whereas *Lactobacillus* treatment partially restored these features. Moreover, the higher network density and clustering coefficient in low-LPS groups suggest increased complexity and potentially greater micro-ecosystem stability in healthy states. Indeed, network complexity is often associated with enhanced ecosystem resilience and stability (22). Compared to the high-LPS group, the low-LPS group exhibited more but smaller modules, indicating a shift toward a small-world network structure. This increased modularity likely represents an adaptive response, allowing the gut community to partition into smaller, resilient subnetworks under AF-induced stress. Such reorganization buffers against species loss and preserves overall network integrity (37). Network stability was assessed by simulating species extinctions via random node removal and targeted hub attacks and computing robustness and natural connectivity. We found that the AF group exhibited lower robustness and natural connectivity than the CK and PRO groups, indicating impaired network stability. Additionally, the network vulnerability in the CK and PRO groups was lower than that observed in the AF group. Moreover, we evaluated cohesion within the networks, which serve as measures of the strength of biotic interactions (22) that influence compositional and network stability. The results showed that cohesion was found to be significantly higher in the CK and PRO groups than in the AF group. Interestingly, cohesion exhibited a significant negative correlation with LPS levels, underscoring the close link between community stability and intestinal LPS burden. Collectively, these findings indicate that microbial networks in low-LPS groups exhibit greater stability than those in the high-LPS group. This reduction in stability may compromise ecological buffering capacity (specifically, the protection against bacterial lysis), thereby underscoring the critical link between microbial network integrity and the containment of intestinal LPS.

Although we have established a link between LPS levels and gut microbiota community stability, the mechanisms by which stability modulates LPS remain unresolved. To address this, we incorporate network module analysis to elucidate the underlying drivers of this relationship. Modules represent densely connected regions in microbial networks (42), reflecting spatial segmentation, niche differentiation, and interspecific relationships within these networks (43). Generally, modules contribute to network stability (37), with the removal of key nodes within a module leading to more substantial changes in the network than if those nodes had remained (44). In our study, we identified a greater number of main modules in the low LPS groups compared to the high LPS group. Notably, we observed that modules featuring *Lactobacillus* as a hub species were prevalent in low LPS groups rather than in the high LPS group. These hub species typically play a critical role in network construction and module formation (44). Notably, most modules correlated positively with *Lactobacillus* abundance, implying its role in module formation. Meanwhile, we also found that these modules were significantly associated with LPS synthetic genes, suggesting that they function as “LPS-reservoir modules” capable of storing LPS and stabilizing it within the modules. We believe that under normal circumstances, the existence of “LPS-reservoir modules” can help stabilize LPS-producing bacteria within them, buffer environmental stress, reduce cell extinction, and thereby decrease LPS release from bacterial lysis. However, we also observed considerable shuffling of species within these modules across different health states. In the AF group, modules that stored LPS in the CK group were transformed into non-main modules or free nodes, which represent more unstable components of the network, increasing the likelihood of strain extinction, replacement, and subsequent release of LPS into the environment. Conversely, in the PRO group, *Lactobacillus* facilitated the reintegration of these LPS producers into the “LPS-reservoir modules,” thereby stabilizing the community. Furthermore, while the number of rare species contributing to the networks exceeded that of abundant species, most modules storing LPS contained a higher proportion of abundant species. The ratio of rare to abundant species (R/A ratio) within the modules was significantly negatively correlated with the relationship between module composition and LPS synthetic genes. This suggests that the module’s capacity to store LPS primarily stems from abundant species, while rare species may play a precarious role within the “LPS-reservoir modules.” Additionally, we found that the correlation between module composition and *Lactobacillus* abundance was significantly negatively correlated with the R/A ratio in the modules, potentially representing a mechanism through which *Lactobacillus* promotes network stability.

### Limitation

This study has certain limitations. Specifically, we relied solely on enzyme-linked immunosorbent assay (ELISA) to quantify LPS levels in both plasma and intestinal contents, without assessing complementary markers such as lipopolysaccharide-binding protein (LBP). Future investigations should incorporate LBP quantification to further enhance the robustness of the findings. Furthermore, the assessment of colonization for the administered *Lactobacillus* strains was restricted to the experimental endpoint. The absence of longitudinal monitoring throughout the 4-week intervention period represents a limitation, as it precludes the characterization of temporal colonization dynamics. Future investigations should incorporate time-series sampling to fully elucidate the dynamic reshaping of the gut microbiota by *Lactobacillus*. While our antibiotic-depleted model demonstrated the dependency of *Lactobacillus* on the resident microbiota, we acknowledge that utilizing germ-free (GF) mice with precise colonization would provide more definitive evidence regarding specific microbial interactions, an avenue that warrants future investigation.

### Conclusion

In conclusion, addressing the limited understanding of the interaction between gut microbiota and AF, this study integrated multiple clinical cohorts to characterize gut microbial dysbiosis in AF patients. Focusing on *Lactobacillus*, a genus markedly altered between patients and healthy individuals, we systematically elucidated, from an ecological perspective, its role in promoting intestinal homeostasis and facilitating the formation of “LPS-reservoir modules,” thereby reducing LPS levels and alleviating AF. These findings offer novel insights into the gut microbiota-AF interaction and suggest new avenues for AF therapy.

## Data Availability

The data sets generated and analyzed during the current study are available in public repositories. The raw 16S rRNA high-throughput sequencing data have been deposited in the NCBI BioProject database under the accession number PRJNA1037604. The metabolomics data are publicly available in the National Genomics Data Center (NGDC) under the accession number PRJCA060467.

## SUPPLEMENTAL MATERIAL

Supplemental figures mbio.00741-26-s0001.pdf Fig. S1 to S10.

Supplemental tables mbio.00741-26-s0002.xlsx Tables S1 to S11.

ASM does not own the copyrights to Supplemental Material that may be linked to, or accessed through, an article. The authors have granted ASM a non-exclusive, world-wide license to publish the Supplemental Material files. Please contact the corresponding author directly for reuse.
