# Supplementary material for: Lactobacillus shapes LPS-reservoir modules within the gut microbiota to mitigate atrial fibrillation
Source: mBio. 2026 May 29;17(7):e00741-26. doi: 10.1128/mbio.00741-26 (PMC13343854; doi:10.1128/mbio.00741-26)
Supplement: Supplemental figures — Fig. S1 to S10. [file mbio.00741-26-s0001.pdf]

Supplementary Information：

Lactobacillus Shapes LPS-Reservoir Modules Within the Gut Microbiota to Mitigate Atrial Fibrillation

Xinyuan Wang[1,2,3#], Xiwen Wang[1,2,6#], Song Xu[1,2*], Jia Zheng[4,5], Yan Pang[1,2,3], Tingting Cheng[1,2,7], Ruiqin Han[1,2,7], Wei Zhao[1,2], Zhiyong Huang[1,2*]

1. Tianjin Key Laboratory for Industrial Biological Systems and Bioprocessing Engineering, Tianjin Institute of Industrial Biotechnology, Chinese Academy of Sciences, Tianjin 300300, China

2. National Technology Innovation Center of Synthetic Biology, Tianjin 300300, China

3. University of Chinese Academy of Sciences, Beijing 100049, China

4. The First Central Clinical School, Tianjin Medical University, Tianjin, China

5. Department of Cardiology, Tianjin First Center Hospital, Tianjin, China

6. State Key Laboratory of Biocatalysis and Enzyme Engineering, School of Life Sciences, Hubei University, Wuhan 430062, Hubei, China

7. College of Bioengineering, Tianjin University of Science and Technology, Tianjin, China

#: The authors marked with “#” are the co-first authors of this article, and both contribute the same amount to this article.

: The authors marked with “” are the co-corresponding authors of this paper,

and they have contributed the same amount to this paper.

Corresponding author

Correspondence to: Song Xu (xu_s@tib.cas.cn) & Zhiyong Huang (huang_zy@tib.cas.cn)

Tianjin Institute of Industrial Biotechnology, Chinese Academy of Sciences

No. 32, West 7th Road

Tianjin Airport Economic Area

Tianjin 300308, P R China

Tel.: +86-22-84861930; Fax: +86-22-84861930

Supplemental Figures legends:

Figure S1. Alpha Diversity in human cohort and Beta Diversity of the 4 Sub-cohorts.

(A) Alpha diversity in human cohort. The red box plot represents the AF group, while the blue box plot represents the CK group.

(B) Beta diversity in the 4 sub-cohorts. The red triangular points represent the CK group, while the black circular points represent the AF group.

(C) Leave-one-dataset-out (LODO) cross-validation performance of the random forest classifier across the 4 sub-cohorts.

(D) Random forest feature importance of the top 30 microbial genera in LODO cross-validation.

Figure S2. Comparison of the relative abundance of *Lactobacillus* in the 4 sub-cohorts.

Figure S3. Comparison of the abundance of *Lactobacillus* in the 4 sub-cohorts and meta-analysis.

(A) Comparison of the abundance of Lactobacillus in the 4 sub-cohorts. The red box plot represents the AF group, while the blue box plot represents the CK group.

(B) Meta-analysis of the 4 sub-cohorts.

Figure S4. Functional pathways and enrichment analysis of metabolites.

(A, B) Functional pathways involved in different metabolites in stools (A) and plasma (B) samples between CK and AF groups.

(C, D) Enrichment analysis of functional pathways involved in different metabolites in stools (C) and plasma (D) samples between CK and AF groups (*$P < 0.05$, **$P < 0.01$, ***$P < 0.001$).

Figure S5. Phylogenetic Tree of Strains and Analysis of Gut Microbiota in Antibiotic Experiments.

(A) A phylogenetic tree constructed from the two *Lactobacillus* strains used in animal experiments and their closest relatives

(B) Correlation networks for the PRO, ABX, and PRO+ABX groups. Nodes represent ASVs, and edges represent correlations. Node size indicates the degree, edge color indicates the direction of correlation, and edge thickness indicates the strength of the correlation.

(C) Cardiac fibrosis area ratio based on Masson staining.

(D) Comparison of the relative abundance of selected opportunistic pathogens in PRO, ABX, and PRO+ABX groups.

(E) Agarose gel electrophoresis validation of qPCR primer specificity.

(F) Melting curve analysis during qPCR quantification.

Figure S6. Three groups of echocardiography and electrocardiogram representation.

(A-D) Cardiac phenotype as measured by echocardiography that LVSd (A), LVPWd (B), LVSs (C), and LVEDV (D). Wilcoxon rank-sum test was used to determine intergroup significance (*n.s*: no significance).

(E) Electrocardiograms of the CK, AF, and PRO groups.

Figure S7. Sequencing depth saturation curves and functional analysis.

(A-B) Rank abundance curves (A) and Rarefaction curves (B) of three groups.

(C) Community structure at the phylum level.

(D) Ternary phase diagram at the genus level.

(E-M) Spearman's correlation between the abundance of gram-negative bacteria in RT taxa and the LPS biosynthesis gene.

(N) Stamp difference analysis of the gene between AF and PRO groups predicted by pricrust2.

Figure S8. Diversity Analysis Excluding *Lactobacillus*.

(A) Comparison of Observed Richness Index among the CK, AF, and PRO groups

(B) Comparison of Rare Taxa among the CK, AF, and PRO groups

(C) Comparison of Beta Diversity among the CK, AF, and PRO groups

(D) Comparison of Alpha Diversity among the CK, AF, and PRO groups

Figure S9. Module hub calculated based on ZiPi analysis in three groups.

Based on ZiPi analysis, the nodes in the network are divided into four categories: peripheral nodes ($Zi < 2.5$, $Pi < 0.62$), connectors ($Zi < 2.5$, $Pi > 0.62$), module hubs ($Zi > 2.5$, $Pi < 0.62$), and network hubs ($Zi > 2.5$, $Pi > 0.62$).

Figure S10. The cohesion within the network is negatively correlated with the LPS level.

Correlation analysis of positive cohesion (A) and negative cohesion (B) with LPS levels.

Figure S1:

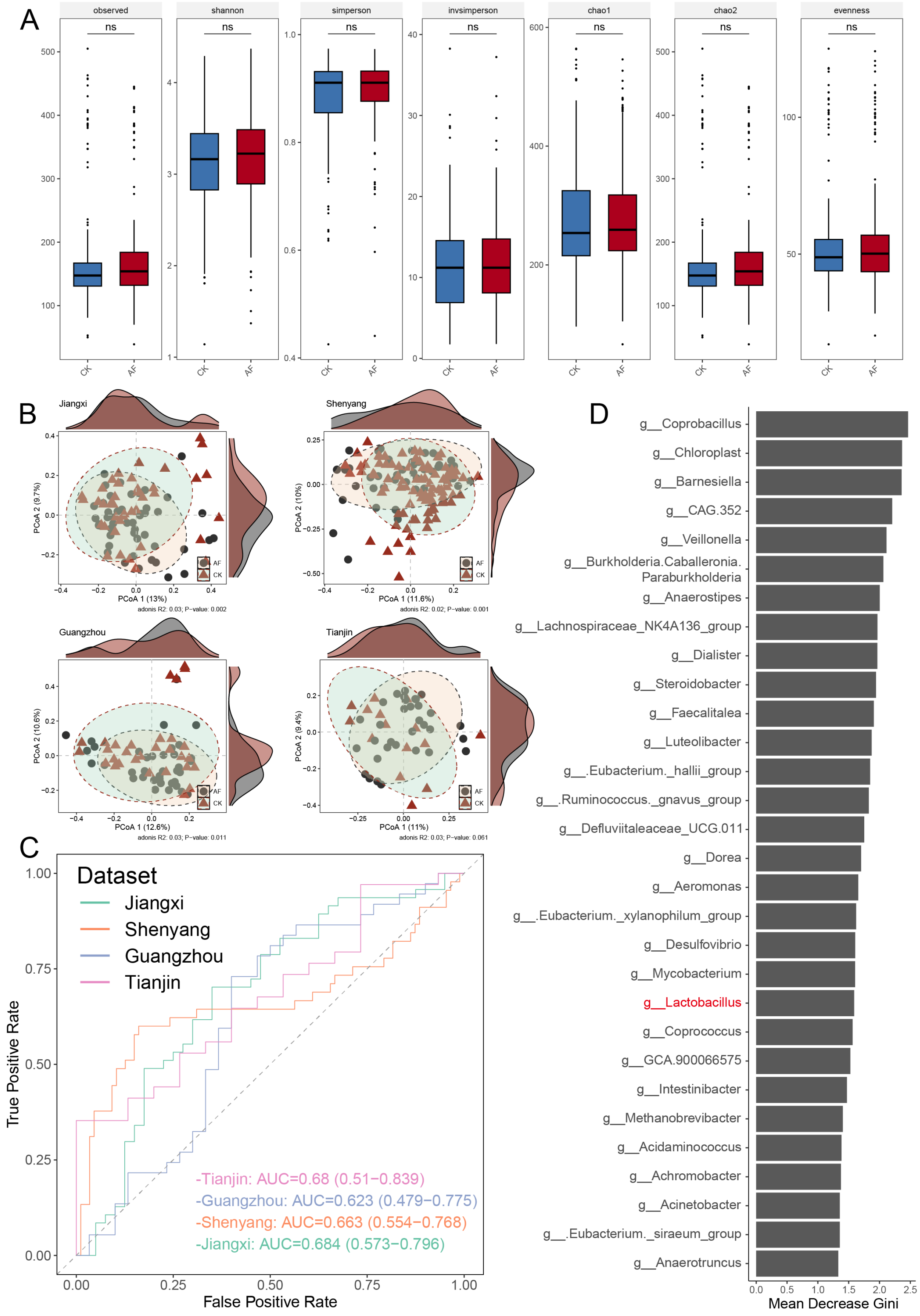
A
observed
shannon
simperson
invsimperson
chao1
chao2
evenness
ns
CK
AF
B
Jiangxi
Shenyang
Guangzhou
Tianjin
PCoA 1 (13%)
PCoA 2 (9.7%)
adonis R2: 0.03; P−value: 0.002
PCoA 1 (11.6%)
PCoA 2 (10%)
adonis R2: 0.02; P−value: 0.001
PCoA 1 (12.6%)
PCoA 2 (10.6%)
adonis R2: 0.03; P−value: 0.011
PCoA 1 (11%)
PCoA 2 (9.4%)
adonis R2: 0.03; P−value: 0.061
C
Dataset
Jiangxi
Shenyang
Guangzhou
Tianjin
True Positive Rate
False Positive Rate
-Tianjin: AUC=0.68 (0.51−0.839)
-Guangzhou: AUC=0.623 (0.479−0.775)
-Shenyang: AUC=0.663 (0.554−0.768)
-Jiangxi: AUC=0.684 (0.573−0.796)
D
g__Coprobacillus
g__Chloroplast
g__Barnesiella
g__CAG.352
g__Veillonella
g__Burkholderia.Caballeronia.Paraburkholderia
g__Anaerostipes
g__Lachnospiraceae_NK4A136_group
g__Dialister
g__Steroidobacter
g__Faecalitalea
g__Luteolibacter
g__.Eubacterium._hallii_group
g__.Ruminococcus._gnavus_group
g__Defluviitaleaceae_UCG.011
g__Dorea
g__Aeromonas
g__.Eubacterium._xylanophilum_group
g__Desulfovibrio
g__Mycobacterium
g__Lactobacillus
g__Coprococcus
g__GCA.900066575
g__Intestinibacter
g__Methanobrevibacter
g__Acidaminococcus
g__Achromobacter
g__Acinetobacter
g__.Eubacterium._siraeum_group
g__Anaerotruncus
Mean Decrease Gini

Figure S2:

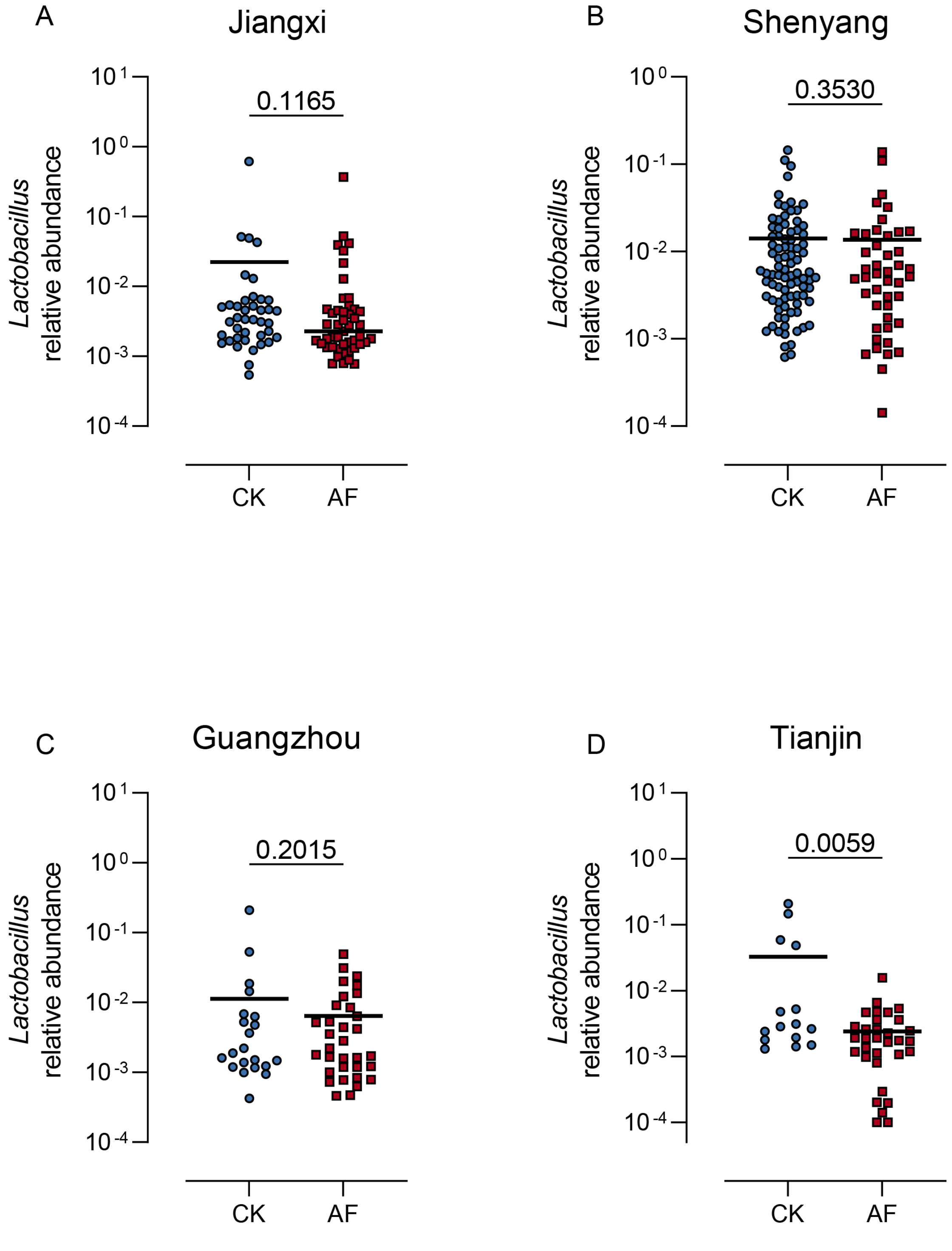
A
Jiangxi
Lactobacillus relative abundance
0.1165
CK
AF
B
Shenyang
Lactobacillus relative abundance
0.3530
CK
AF
C
Guangzhou
Lactobacillus relative abundance
0.2015
CK
AF
D
Tianjin
Lactobacillus relative abundance
0.0059
CK
AF

Figure S3:

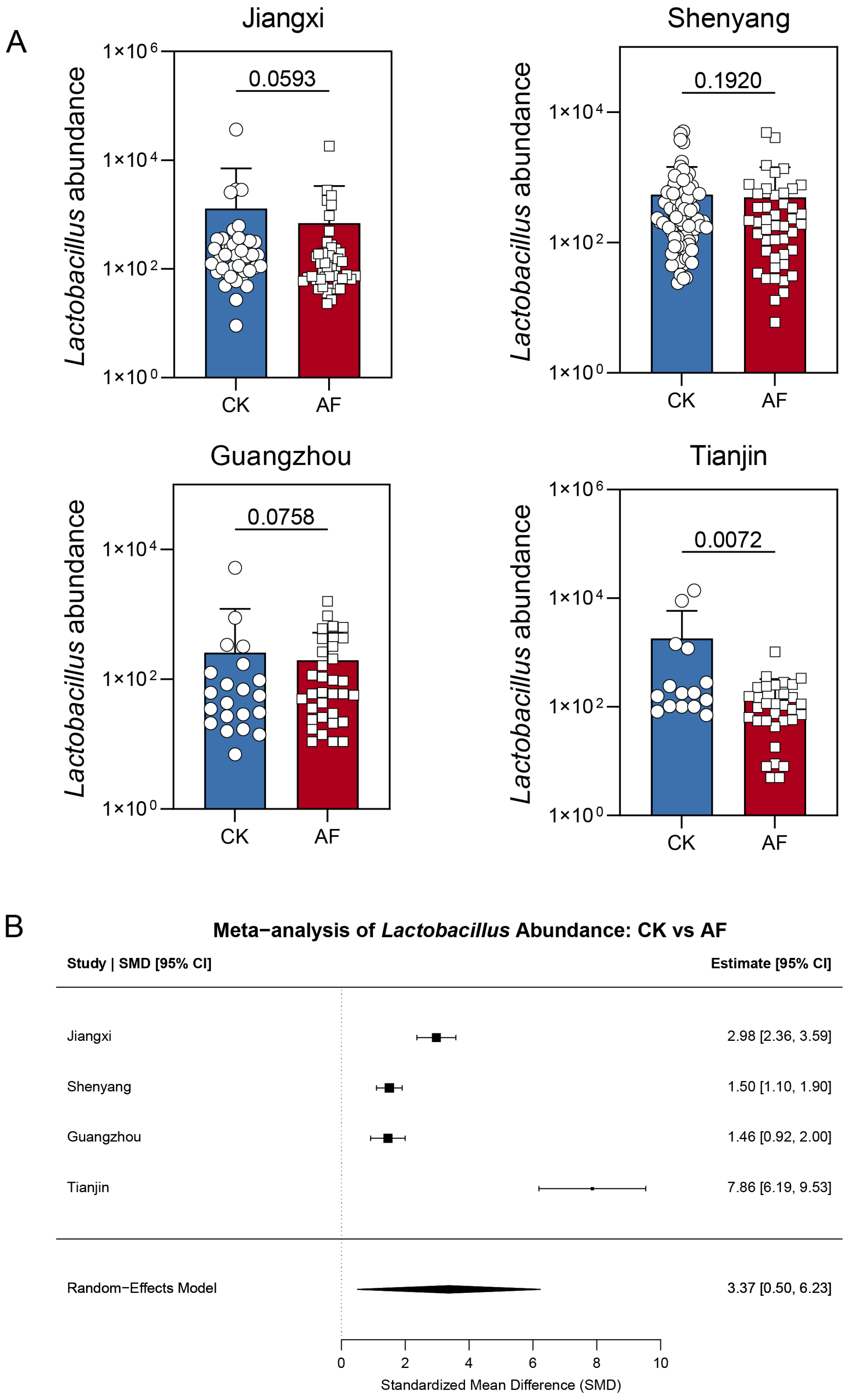
A
Jiangxi
0.0593
Lactobacillus abundance
1×10^6
1×10^4
1×10^2
1×10^0
CK
AF
Shenyang
0.1920
Guangzhou
0.0758
Tianjin
0.0072
B
Meta−analysis of Lactobacillus Abundance: CK vs AF
Study | SMD [95% CI]
Estimate [95% CI]
Jiangxi
2.98 [2.36, 3.59]
Shenyang
1.50 [1.10, 1.90]
Guangzhou
1.46 [0.92, 2.00]
Tianjin
7.86 [6.19, 9.53]
Random−Effects Model
3.37 [0.50, 6.23]
0
2
4
6
8
10
Standardized Mean Difference (SMD)

Figure S4:

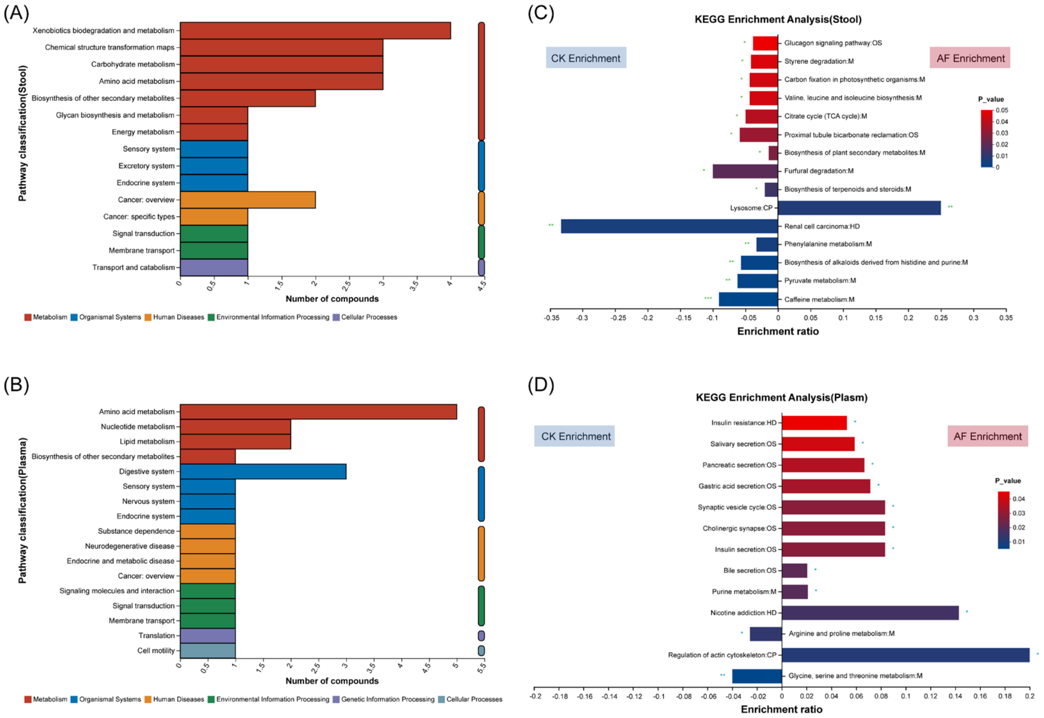

(A)
Pathway classification(Stool)
Xenobiotics biodegradation and metabolism
Chemical structure transformation maps
Carbohydrate metabolism
Amino acid metabolism
Biosynthesis of other secondary metabolites
Glycan biosynthesis and metabolism
Energy metabolism
Sensory system
Excretory system
Endocrine system
Cancer: overview
Cancer: specific types
Signal transduction
Membrane transport
Transport and catabolism
0 0.5 1 1.5 2 2.5 3 3.5 4 4.5
Number of compounds
Metabolism Organismal Systems Human Diseases Environmental Information Processing Cellular Processes
(C)
KEGG Enrichment Analysis(Stool)
CK Enrichment
AF Enrichment
Glucagon signaling pathway:OS
Styrene degradation:M
Carbon fixation in photosynthetic organisms:M
Valine, leucine and isoleucine biosynthesis:M
Citrate cycle (TCA cycle):M
Proximal tubule bicarbonate reclamation:OS
Biosynthesis of plant secondary metabolites:M
Furfural degradation:M
Biosynthesis of terpenoids and steroids:M
Lysosome:CP
Renal cell carcinoma:HD
Phenylalanine metabolism:M
Biosynthesis of alkaloids derived from histidine and purine:M
Pyruvate metabolism:M
Caffeine metabolism:M
P_value
0.05 0.04 0.03 0.02 0.01 0
-0.35 -0.3 -0.25 -0.2 -0.15 -0.1 -0.05 0 0.05 0.1 0.15 0.2 0.25 0.3 0.35
Enrichment ratio
(B)
Pathway classification(Plasma)
Amino acid metabolism
Nucleotide metabolism
Lipid metabolism
Biosynthesis of other secondary metabolites
Digestive system
Sensory system
Nervous system
Endocrine system
Substance dependence
Neurodegenerative disease
Endocrine and metabolic disease
Cancer: overview
Signaling molecules and interaction
Signal transduction
Membrane transport
Translation
Cell motility
0 0.5 1 1.5 2 2.5 3 3.5 4 4.5 5 5.5
Number of compounds
Metabolism Organismal Systems Human Diseases Environmental Information Processing Genetic Information Processing Cellular Processes
(D)
KEGG Enrichment Analysis(Plasm)
CK Enrichment
AF Enrichment
Insulin resistance:HD
Salivary secretion:OS
Pancreatic secretion:OS
Gastric acid secretion:OS
Synaptic vesicle cycle:OS
Cholinergic synapse:OS
Insulin secretion:OS
Bile secretion:OS
Purine metabolism:M
Nicotine addiction:HD
Arginine and proline metabolism:M
Regulation of actin cytoskeleton:CP
Glycine, serine and threonine metabolism:M
P_value
0.04 0.03 0.02 0.01
-0.2 -0.18 -0.16 -0.14 -0.12 -0.1 -0.08 -0.06 -0.04 -0.02 0 0.02 0.04 0.06 0.08 0.1 0.12 0.14 0.16 0.18 0.2
Enrichment ratio

Figure S5:

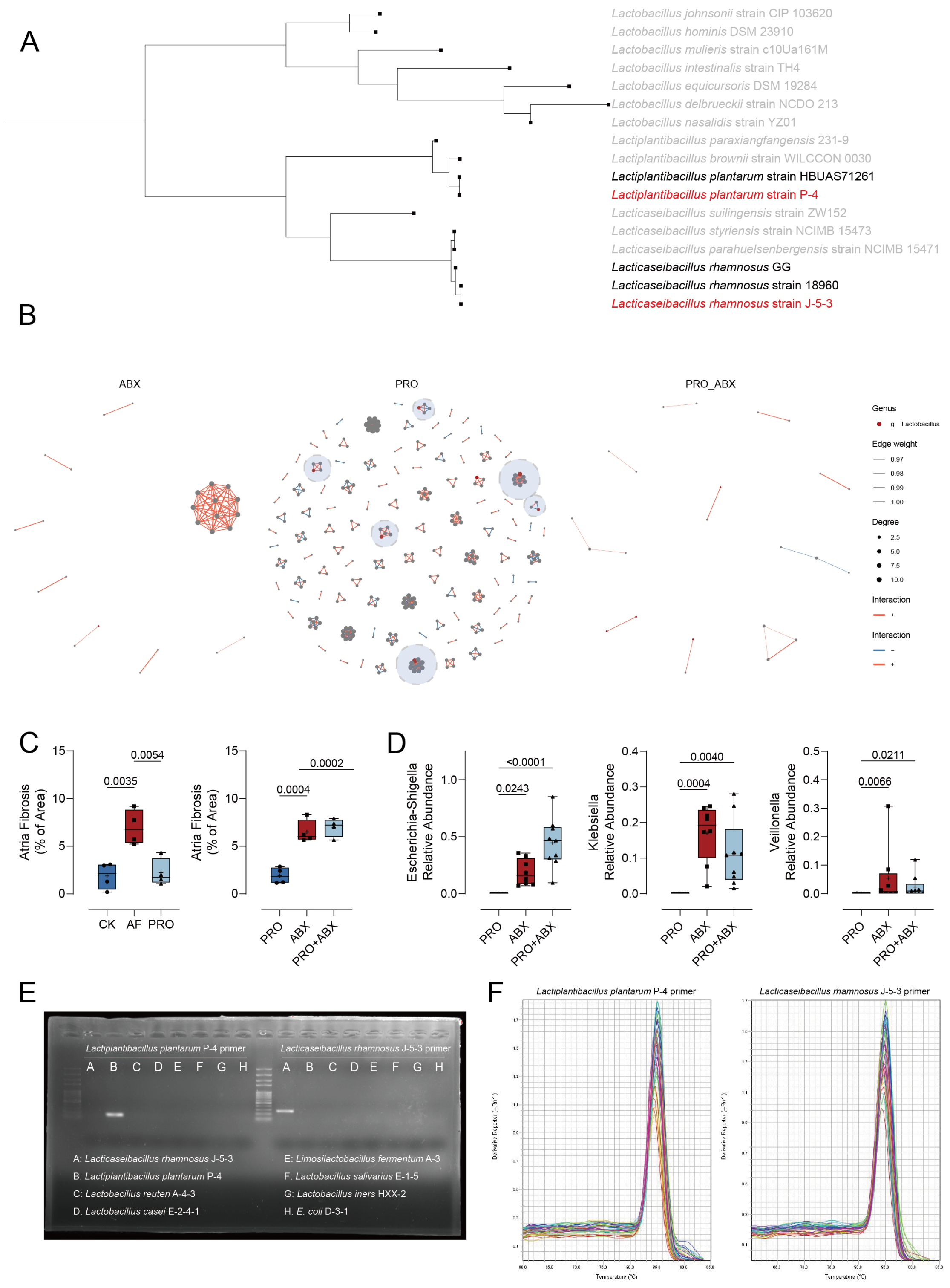
A
Lactobacillus johnsonii strain CIP 103620
Lactobacillus hominis DSM 23910
Lactobacillus mulieris strain c10Ua161M
Lactobacillus intestinalis strain TH4
Lactobacillus equicursoris DSM 19284
Lactobacillus delbrueckii strain NCDO 213
Lactobacillus nasalidis strain YZ01
Lactiplantibacillus paraxiangfangensis 231-9
Lactiplantibacillus brownii strain WILCCON 0030
Lactiplantibacillus plantarum strain HBUAS71261
Lactiplantibacillus plantarum strain P-4
Lacticaseibacillus suilingensis strain ZW152
Lacticaseibacillus styriensis strain NCIMB 15473
Lacticaseibacillus parahuelsenbergensis strain NCIMB 15471
Lacticaseibacillus rhamnosus GG
Lacticaseibacillus rhamnosus strain 18960
Lacticaseibacillus rhamnosus strain J-5-3
B
ABX
PRO
PRO_ABX
Genus
g__Lactobacillus
Edge weight
0.97
0.98
0.99
1.00
Degree
2.5
5.0
7.5
10.0
Interaction
+
Interaction
-
+
C
Atria Fibrosis (% of Area)
CK
AF
PRO
0.0035
0.0054
PRO
ABX
PRO+ABX
0.0004
0.0002
D
Escherichia-Shigella Relative Abundance
0.0243
<0.0001
Klebsiella Relative Abundance
0.0004
0.0040
Veillonella Relative Abundance
0.0066
0.0211
E
Lactiplantibacillus plantarum P-4 primer
Lacticaseibacillus rhamnosus J-5-3 primer
A B C D E F G H
A: Lacticaseibacillus rhamnosus J-5-3
B: Lactiplantibacillus plantarum P-4
C: Lactobacillus reuteri A-4-3
D: Lactobacillus casei E-2-4-1
E: Limosilactobacillus fermentum A-3
F: Lactobacillus salivarius E-1-5
G: Lactobacillus iners HXX-2
H: E. coli D-3-1
F
Lactiplantibacillus plantarum P-4 primer
Lacticaseibacillus rhamnosus J-5-3 primer
Derivative Reporter (-Rn')
Temperature (°C)

Figure S6:

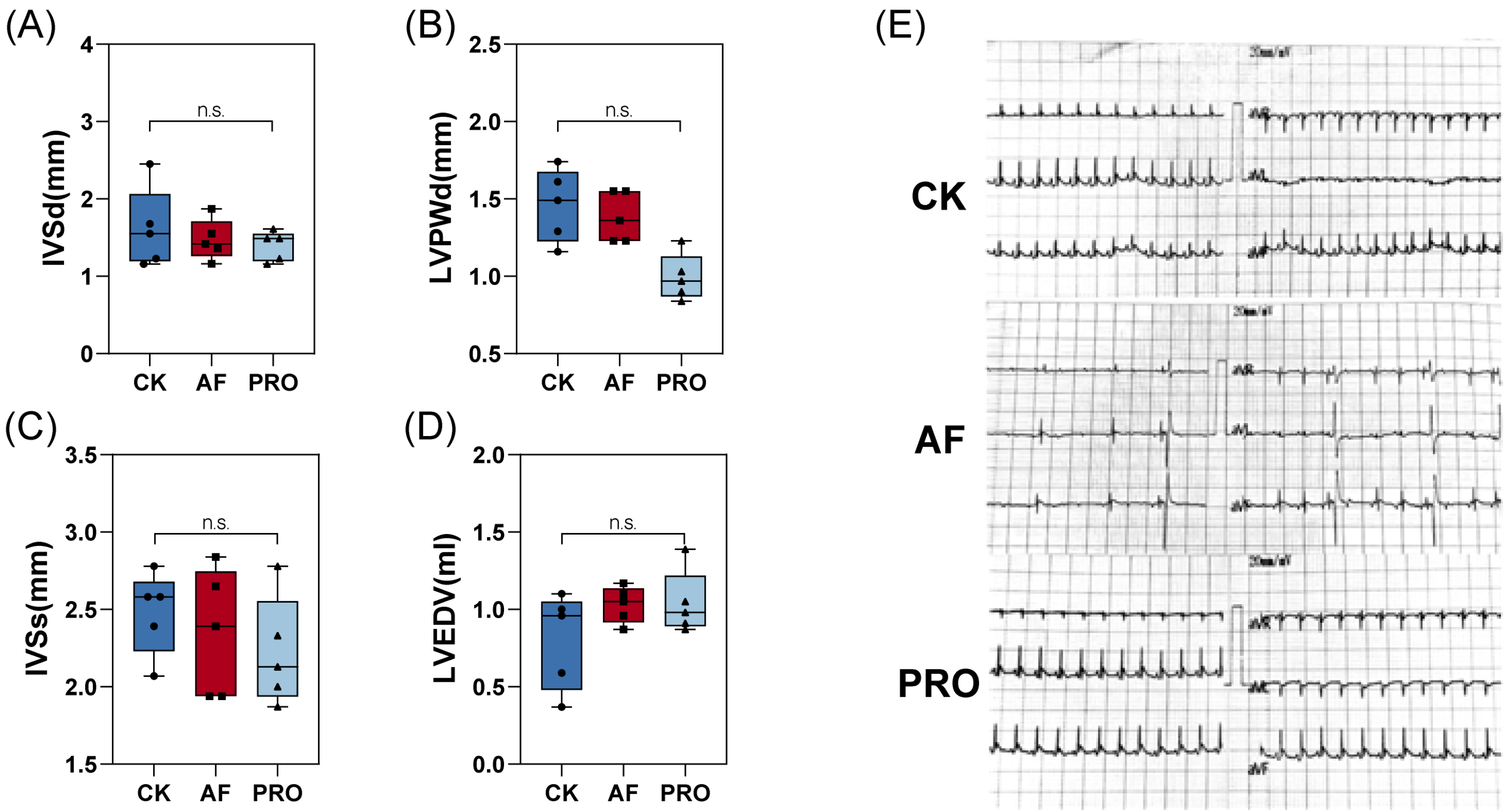
(A)
IVSd(mm)
n.s.
CK
AF
PRO
(B)
LVPWd(mm)
n.s.
CK
AF
PRO
(C)
IVSs(mm)
n.s.
CK
AF
PRO
(D)
LVEDV(ml)
n.s.
CK
AF
PRO
(E)
CK
AF
PRO

Figure S7:

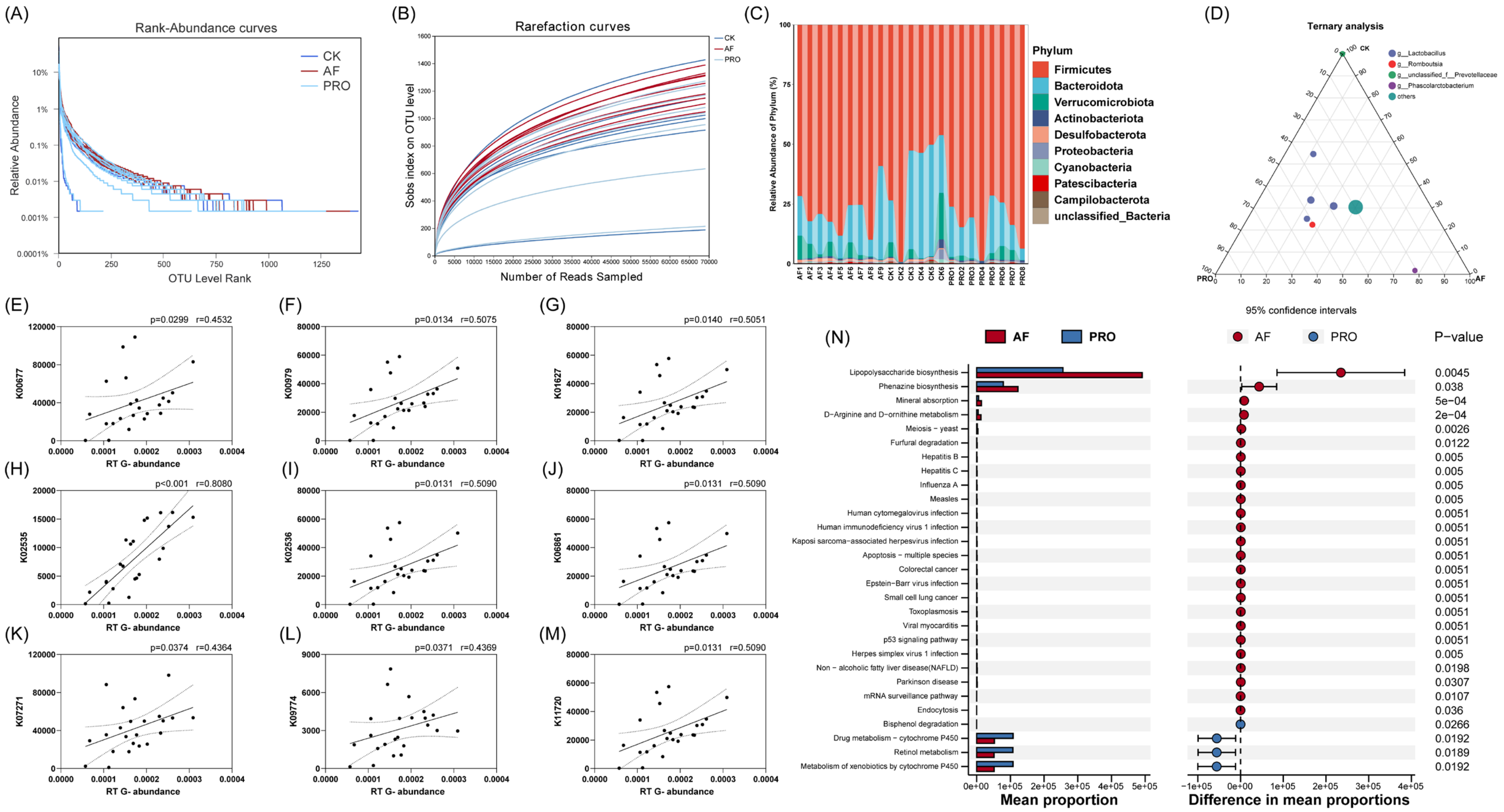

(A)
Rank-Abundance curves
CK
AF
PRO
Relative Abundance
OTU Level Rank
(B)
Rarefaction curves
Sobs index on OTU level
Number of Reads Sampled
(C)
Relative Abundance of Phylum (%)
Phylum
Firmicutes
Bacteroidota
Verrucomicrobiota
Actinobacteriota
Desulfobacterota
Proteobacteria
Cyanobacteria
Patescibacteria
Campilobacterota
unclassified_Bacteria
(D)
Ternary analysis
(E)
p=0.0299 r=0.4532
K00677
RT G- abundance
(F)
p=0.0134 r=0.5075
K00979
(G)
p=0.0140 r=0.5051
K01627
(H)
p<0.001 r=0.8080
K02535
(I)
p=0.0131 r=0.5090
K02536
(J)
p=0.0131 r=0.5090
K06861
(K)
p=0.0374 r=0.4364
K07271
(L)
p=0.0371 r=0.4369
K09774
(M)
p=0.0131 r=0.5090
K11720
(N)
AF
PRO
95% confidence intervals
P-value
Lipopolysaccharide biosynthesis 0.0045
Phenazine biosynthesis 0.038
Mineral absorption 5e−04
D−Arginine and D−ornithine metabolism 2e−04
Meiosis − yeast 0.0026
Furfural degradation 0.0122
Hepatitis B 0.005
Hepatitis C 0.005
Influenza A 0.005
Measles 0.005
Human cytomegalovirus infection 0.0051
Human immunodeficiency virus 1 infection 0.0051
Kaposi sarcoma−associated herpesvirus infection 0.0051
Apoptosis − multiple species 0.0051
Colorectal cancer 0.0051
Epstein−Barr virus infection 0.0051
Small cell lung cancer 0.0051
Toxoplasmosis 0.0051
Viral myocarditis 0.0051
p53 signaling pathway 0.0051
Herpes simplex virus 1 infection 0.005
Non − alcoholic fatty liver disease(NAFLD) 0.0198
Parkinson disease 0.0307
mRNA surveillance pathway 0.0107
Endocytosis 0.036
Bisphenol degradation 0.0266
Drug metabolism − cytochrome P450 0.0192
Retinol metabolism 0.0189
Metabolism of xenobiotics by cytochrome P450 0.0192
Mean proportion
Difference in mean proportions

Figure S8:

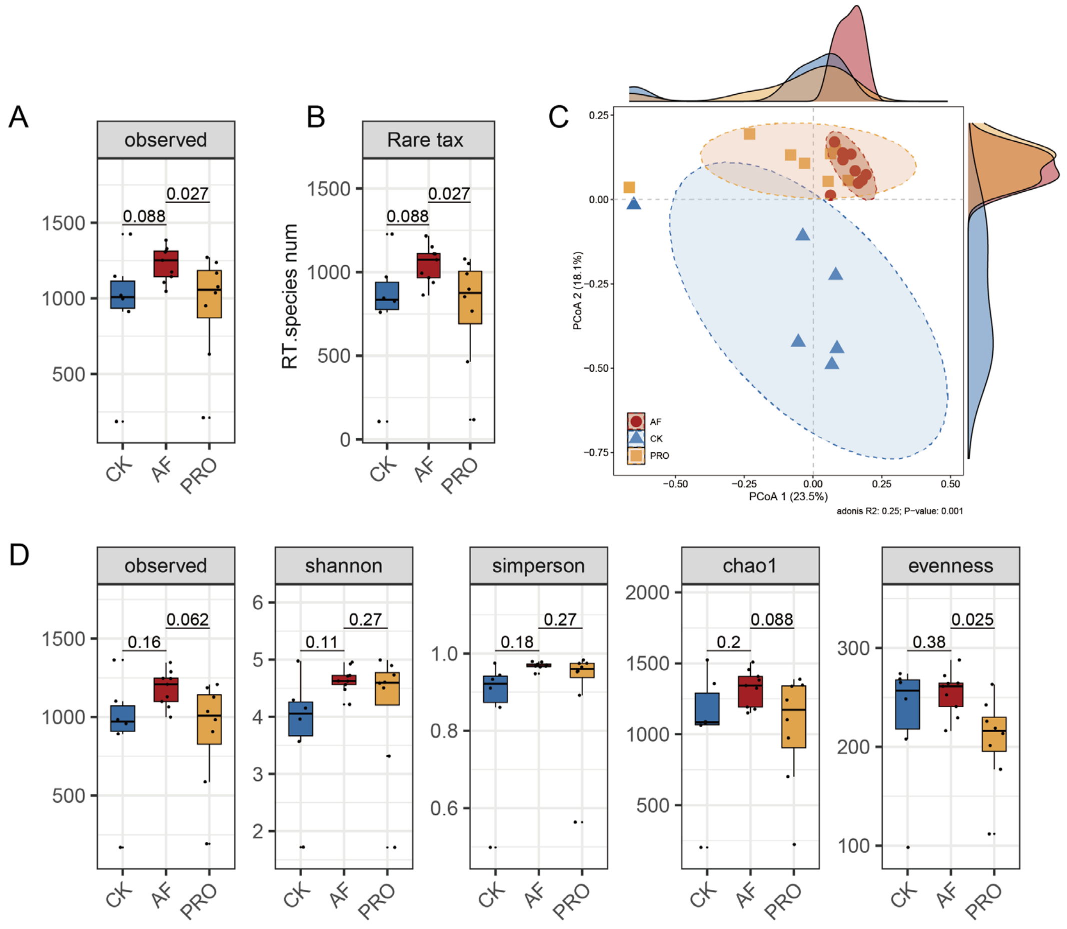
A
observed
0.088
0.027
1500
1000
500
CK
AF
PRO
B
Rare tax
RT.species num
0.088
0.027
1500
1000
500
0
CK
AF
PRO
C
PCoA 2 (18.1%)
PCoA 1 (23.5%)
0.25
0.00
−0.25
−0.50
−0.75
−0.50
−0.25
0.00
0.25
0.50
AF
CK
PRO
adonis R2: 0.25; P-value: 0.001
D
observed
0.16
0.062
shannon
0.11
0.27
simperson
0.18
0.27
chao1
0.2
0.088
2000
evenness
0.38
0.025
300
200
100
CK
AF
PRO

Figure S9:

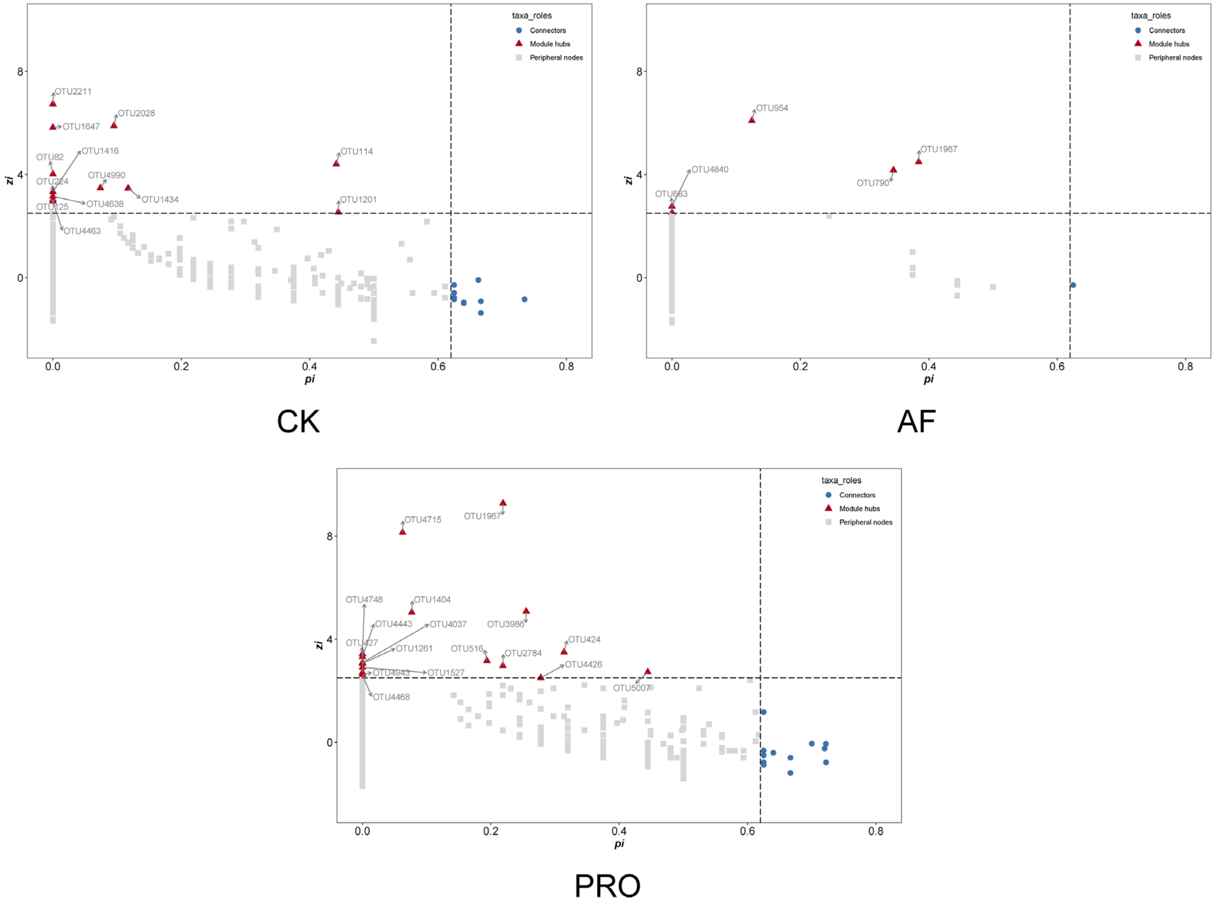
taxa_roles
Connectors
Module hubs
Peripheral nodes
zi
pi
OTU2211
OTU1647
OTU2028
OTU1416
OTU82
OTU114
OTU4990
OTU1434
OTU4638
OTU1201
OTU4463
CK
OTU954
OTU4840
OTU1967
OTU790
AF
OTU4715
OTU1967
OTU4748
OTU1404
OTU4443
OTU4037
OTU3986
OTU1261
OTU516
OTU424
OTU2784
OTU4426
OTU1527
OTU5007
OTU4468
PRO

Figure S10:

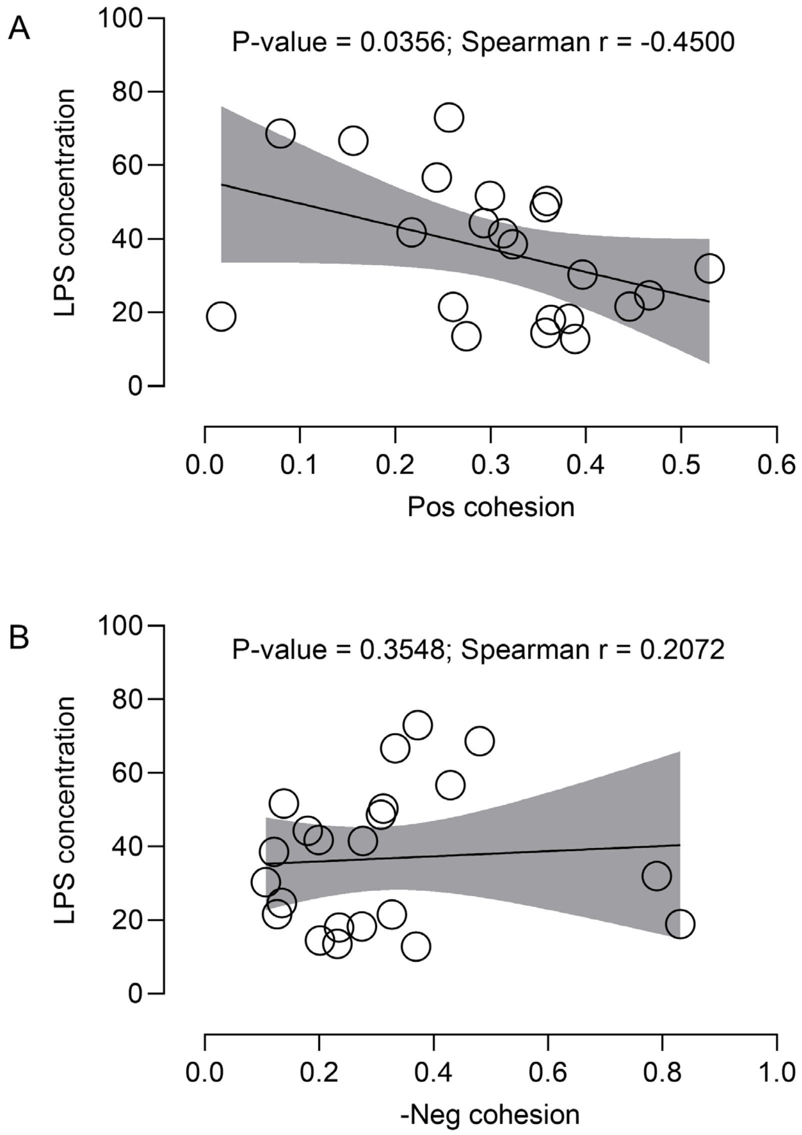
A
P-value = 0.0356; Spearman r = -0.4500
LPS concentration
100
80
60
40
20
0
0.0
0.1
0.2
0.3
0.4
0.5
0.6
Pos cohesion
B
P-value = 0.3548; Spearman r = 0.2072
LPS concentration
100
80
60
40
20
0
0.0
0.2
0.4
0.6
0.8
1.0
-Neg cohesion
